# Adélie penguins foraging consistency and site fidelity are conditioned by breeding status and environmental conditions

**DOI:** 10.1371/journal.pone.0244298

**Published:** 2021-01-22

**Authors:** Candice Michelot, Akiko Kato, Thierry Raclot, Yan Ropert-Coudert

**Affiliations:** 1 Centre d’Etudes Biologiques de Chizé, La Rochelle Université–CNRS, UMR 7372, Villiers en Bois, France; 2 Institut Pluridisciplinaire Hubert Curien–CNRS, UMR 7178, Strasbourg, France; MARE – Marine and Environmental Sciences Centre, PORTUGAL

## Abstract

There is a growing interest in studying consistency and site fidelity of individuals to assess, respectively, how individual behaviour shapes the population response to environmental changes, and to highlight the critical habitats needed by species. In Antarctica, the foraging activity of central place foragers like Adélie penguins (*Pygoscelis adeliae*) is constrained by the sea-ice cover during the breeding season. We estimated the population-level repeatability in foraging trip parameters and sea-ice conditions encountered by birds across successive trips over several years, and we examined their foraging site fidelity linked to sea-ice concentrations throughout the chick-rearing season. Penguins’ foraging activity was repeatable despite varying annual sea-ice conditions. Birds’ site fidelity is constrained by both sea-ice conditions around the colony that limit movements and resources availability, and also behavioural repeatability of individuals driven by phenological constraints. Adélie penguins favoured sea-ice concentrations between 20–30%, as these facilitate access to open water while opening multiple patches for exploration in restricted areas in case of prey depletion. When the sea-ice concentration became greater than 30%, foraging site fidelity decreased and showed higher variability, while it increased again after 60%. Between two trips, the foraging site fidelity remained high when sea-ice concentration changed by ± 10% but showed greater variability when sea-ice concentrations differed on a larger range. In summary, Adélie penguins specialize their foraging behaviour during chick-rearing according to sea-ice conditions to enhance their reproductive success. The balance between being consistent under favourable environmental conditions vs. being flexible under more challenging conditions may be key to improving foraging efficiency and reproductive success to face fast environmental changes.

## 1. Introduction

The marine environment is highly dynamic. Although direct observations of predation are rarely possible, foraging behaviour of predators such as seabirds is often used as an indicator of resource-enriched areas and prey availability [1,2]. Seabirds are central-place foraging species: they commute regularly between their nest on land to incubate eggs or feed their chicks during the breeding season, and the sea where they forage [3]. Although seabirds’ breeding season mostly matches the peak of food availability [4], the distribution of prey through space and time can vary according to the environmental conditions.

High plasticity in phenological responses under a changing environment is thought to improve a species’ breeding success and increased their fitness [1]. Flexibility in foraging behaviour is thus an important trait to cope with changes in the environment [5,6]. However, adopting flexible behaviour may not be effective to buffer against extreme environmental conditions and highly scarce resources when animals are phenologically constrained [7,8]. Conversely, some mechanisms such as individual consistency may have been adopted to limit intraspecific competition [9–11]. Foraging strategies are thought to differ according to several factors such as the age and the sex, or the environmental conditions and previous experiences [8,12–14]. As such, a population can include individuals with greater consistency in their foraging behaviour than others.

In dynamic environments, individual’s behavioural consistency indicates how it copes with constraints, for example, during the succession of foraging trips throughout a breeding season [13,15]. To this end, several types of measures can be used to assess individuals’ behavioural consistency. Firstly, repeatability indices measure the degree of consistency, i.e. the fraction of behavioural variation that can be attributed to differences between individuals. A repeatable behaviour indicates a low within-individual variance after several measures compared to inter-individual variance [16–18]. Secondly, spatial similarity in foraging areas visited (or foraging site fidelity) assesses whether individuals rely on a given set of specific environmental conditions and can inform on the distribution of sustainable foraging grounds and prey availability [13]. As such, individual consistency in foraging behaviour can highlight specialization, i.e. the use of a subset of the available resources [9,11] in a given foraging area or, conversely, the degree of flexibility that an individual displays in its environment. The behavioural consistency in foraging activity can originate from heritable phenotypes, be transmitted socially, or be the result of individual decision-making based on previous experiences [12,16,19–21].

In Antarctica, Adélie penguins (*Pygoscelis adeliae*) are good models to study the effect of spatial and temporal environmental variations on the consistency or flexibility of their foraging behaviour, as well as on their foraging site fidelity. Their foraging success is highly dependent on sea-ice conditions. Sea-ice conditions vary significantly during a breeding season, leading to a highly dynamic foraging environment in terms of prey availability [22]. When birds arrive at the colony at the beginning of the austral summer, the sea-ice extent is generally high. Incubating penguins target the sea-ice edge, a productive and predictable area for prey abundance [22–24], where they mainly feed on Antarctic krill (*Euphausia superba*) [25]. Later, sea ice recedes and the resources become available closer to the colony, at the period when penguins need to perform shorter trips to feed their growing chicks [26,27]. At that time, penguins feed mostly on ice krill (*E*. *crystallorophias*) and juveniles of Antarctic silverfish (*Pleuragramma antarctica*) in the more neritic waters of the continental shelf [25,28]. However, the sea-ice scape varies greatly during this period of the breeding cycle, affecting the distribution of resources and consequently this may affect penguins’ foraging strategies [29,30].

In East Antarctica, sea-ice cover along the coastline has increased in recent years [31,32]. Recently, the colony on the Ile des Pétrels (Terre Adélie) experienced two massive breeding failures due to extreme environmental events, including high sea-ice extent and persistence around the colony during the breeding season [30,33]. Adult Adélie penguins performed longer trips than usual and/or did not find enough resources around the colony for provisioning their chicks [30]. Under such variable conditions–both within and between years–it is thus important to assess the plasticity of Adélie penguins’ foraging behaviour.

Here, we aimed to study the repeatability in Adélie penguins’ foraging trip parameters during successive chick-rearing trips across years, when birds experienced contrasted sea-ice scapes. We also aimed to assess foraging site fidelity between trips, and relate these to environmental conditions and their spatio-temporal variations within and across years. The Adélie penguins’ repeatability has–to the best of our knowledge–only been studied in terms of their diving behaviour [34].

Our main hypothesis was that repeatability in foraging efforts and foraging site fidelity should be high under optimal conditions. Optimal sea-ice conditions for Adélie penguins are thought to be when the sea-ice concentration is around 20–30%, i.e. a state of diffuse sea-ice in which food availability is expected to be high, leading to enhanced foraging success [35–38]. In such concentrations around the colony (facilitating transit movements for birds), and with small sea-ice scape variation from one trip to another, we thus expect birds to easily access suitable foraging grounds and perform short foraging trips back and forth to the same zone they previously visited.

Inversely, under more challenging conditions (high sea-ice concentration and large extent around the colony), sea-ice cover constrains birds from traveling farther. We expect penguins to return to the same foraging grounds around the colony until prey depletion occurs, from which point we expect them to expand their exploration range to find new profitable foraging areas, thereby increasing their foraging effort [36,38,39].

## 2. Material—Methods

### 2.1. Foraging activity data collection and processing

**Fig 1:**
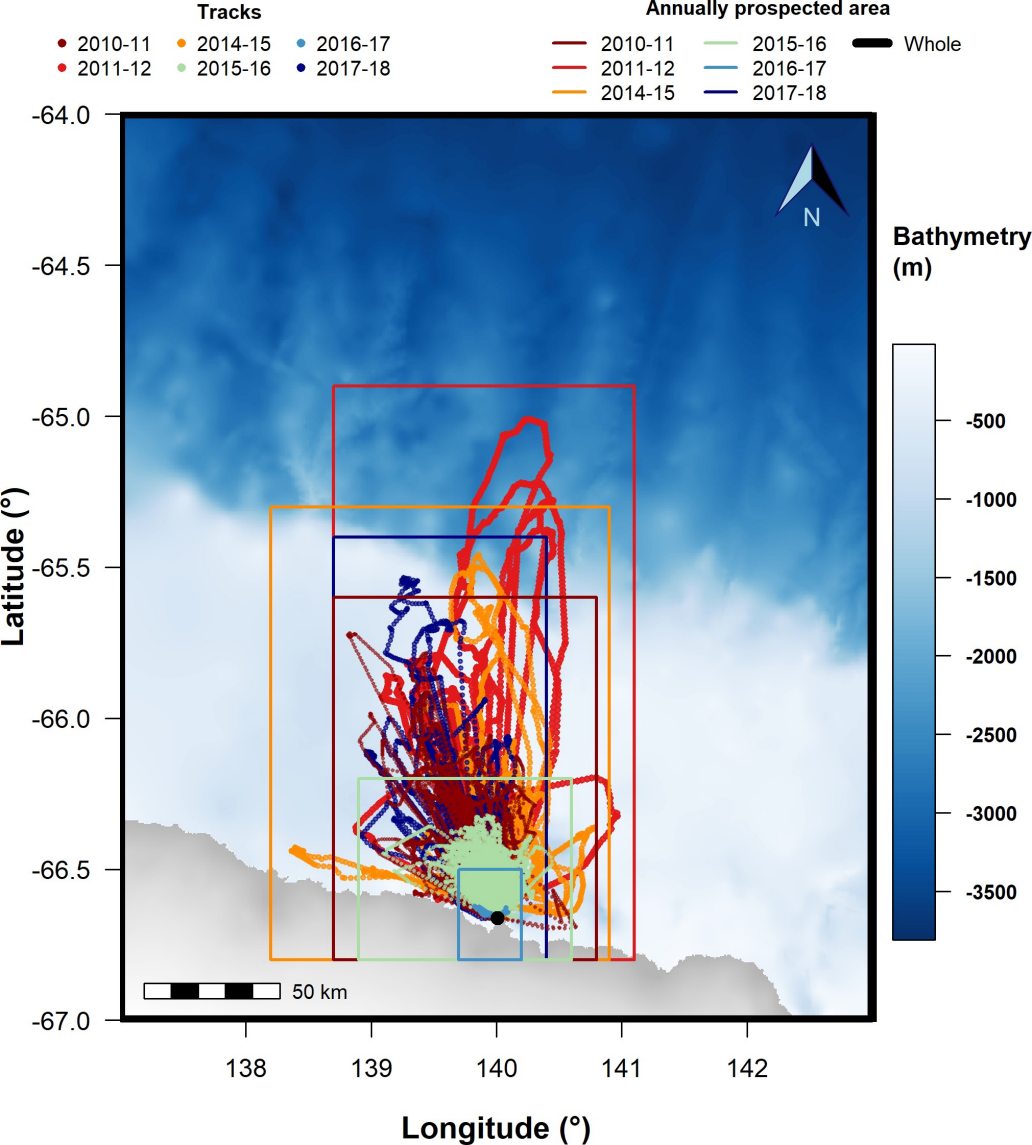
Map representing the location of the colony (black dot), all the annual birds’ trips kept in the analysis as dots (color code per year), the limits of the annually prospected areas (same color code as annual birds’ trips) and of the whole study area (black). Note that the color code for each year will be kept in other figures.

This study was conducted in Terre Adélie (East Antarctica), on the Ile des Pétrels, near Dumont d’Urville French station (140.01° E, 66.66° S). This study was approved by the ethic committee of the Terres Australes et Antarctiques Françaises (TAAF) and the French regional ethic committee 54. Adélie penguins were equipped with GPS loggers over several successive trips during the chick-rearing season (late December to mid-January), from 2010–11 to 2017–18 (except 2012–13 and 2013–14) (Fig 1, Table 1). Breeders (i.e. adults rearing one or two chicks) were captured at their nest and devices were attached to the birds’ back feathers with marine tape, mastic and secured with cable-ties [40]. Several types of GPS were used according to years: CatTrack and CatLog (Catnip Technologies, USA), ca. 14 x 35 x 70 mm, 30 g, customized to be waterproof, and AxyTrek (Technosmart Europe srl, Italy), ca. 10 x 25 x 40 mm, 25 g (the heaviest logger representing less than 1% of the body mass of the lightest equipped bird), were programmed to record a location every 1 to 15 minutes depending on loggers, years and battery capacity. Birds were then released at their nest. When at sea, birds’ nests were monitored from a distance every 2–3 hours. Tagged birds were recaptured on their nest after several consecutive trips to retrieve the loggers. A total of 129 birds with two to nine trips recorded (depending on the bird and year, with an average of 2.46 ± 1.69 trips recorded per bird, see S1 Table in S2 File), representing 389 trips, were used in the analyses (Table 1). Note that we cannot ascertain that the birds captured were different from one year to another. Yet, we tried every year to equip birds on different nests in the colony of > 15 000 breeding pairs so that there is a low chance to recapture the same birds.

**Table 1:** Annual number of individuals (N_ind_) tracked during a given number of trips (N_trip_), total number of individuals (N_ind_ total) and trips (N_trip_ total) per year, and total number of individuals tracked and trips used for that study after removing incomplete tracks.

	Year						Total
	2010–2011	2011–2012	2014–2015	2015–2016	2016–2017	2017–2018	
**N**_**ind**_ **(N**_**trip**_ **= 2)**	3	5	11	36	2	19	76
**N**_**ind**_ **(N**_**trip**_ **= 3)**	6	1	_-_	14	_-_	_-_	21
**N**_**ind**_ **(N**_**trip**_ **= 4)**	4	1	_-_	7	_-_	_-_	12
**N**_**ind**_ **(N**_**trip**_ **= 5)**	4	_-_	_-_	7	_-_	_-_	11
**N**_**ind**_ **(N**_**trip**_ **= 6)**	_-_	_-_	_-_	1	_-_	_-_	1
**N**_**ind**_ **(N**_**trip**_ **= 7)**	_-_	_-_	_-_	2	_-_	_-_	2
**N**_**ind**_ **(N**_**trip**_ **= 8)**	_-_	_-_	_-_	3	_-_	_-_	3
**N**_**ind**_ **(N**_**trip**_ **= 9)**	_-_	_-_	_-_	3	_-_	_-_	3
**N**_**ind**_ **total**	**17**	**7**	**11**	**73**	2*	**19**	**129**
**N**_**trip**_ **total**	**60**	**17**	**22**	**248**	**4**	**38**	**389**

* Note the low number of equipped penguins in 2016–17: this is because it is difficult to instrument many individuals in years with poor environmental conditions (see in discussion) due to the higher rate of breeding failure compared with years with better prey availability.

‘-‘ stands for no trip recorded.

We calculated the peak hatching date from the Adélie penguin colony, as the median date of hatching where 50% of the first laid egg have hatched (S2 Table in S2 File). We then calculated the time elapsed between the peak hatching date and the departure date of each trip for each equipped bird.

The GPS tracks collected were processed using R [41]. Erroneous location with missing date-time or recorded in the Northern Hemisphere were removed. Duplicated points (two points recorded at the same location and same date-time) were removed, and outlier locations based on unrealistic speed were removed with a 10 km.h^- 1^ speed filter (Adélie penguins swim on average at 2.0 m.s^-1^ = 7.2 km.h^-1^, [42,43]). Excessive points recorded at the colony before the departure and after the return of birds were removed manually. For incomplete tracks (i.e. tracks for which the GPS stopped recording when the birds were still at sea), the total trip duration was calculated between the date-time of the departure from the colony recorded by the GPS and the returning date-time recorded during the routine nest monitoring. Incomplete tracks with > 70% of the trip duration recorded, covering most of the foraging phase of the trip (see Material and Methods 2.4.) were completed by adding a virtual point at the colony with the date and time of the birds return recorded during the routine checks. Incomplete tracks with < 70% of the trip duration recorded were discarded from the analysis.

GPS tracks were resampled with a regular time step of 20 minutes between locations, using the *redisltraj()* function from the “adehabitatLT” R package [44]. For each individual, the number of trips, the initial and final date-time of each trip (UTC), and four parameters representing the foraging effort were calculated: *i)* the total trip duration, *ii)* the total trip distance (i.e. the cumulative horizontal distance between all GPS locations per bird per trip), *iii)* the maximal distance from the colony (i.e. the straight line distance between the colony and the most distal point of a trip) and *iv)* the heading (bearing angle) between the colony and the most distal point of a trip (see S1 Table in S2 File for individual details).

### 2.2. Sea-ice concentration and foraging trip range

Daily sea-ice concentration data (Advanced Microwave Scanning Radiometer, AMSR and AMSR-2, grid cell resolution of 6.25 km) were downloaded from the website of the University of Bremen, Germany (https://seaice.uni-bremen.de/data/amsr2/asi_daygrid_swath), between the starting and returning date of each bird’s trip for all years. In 2012, AMSR data were not available. We thus downloaded daily satellite imagery data collected via SSM/I (ftp://ftp.ifremer.fr/ifremer/cersat/products/gridded/psi-concentration/data/antarctic/daily) with a 12.5 km grid cell resolution, for all years.

For each bird’s trip, daily sea-ice concentration data were extracted and transformed into raster maps with the “raster” R package, with the two types of data at both 6.25 and 12.5 km grid cell resolution (except 2012 for which 6.25 km resolution was not available). Each GPS location was associated with the relevant daily sea-ice concentration in the given cell at the two resolutions. For all years except 2012, we compared the values at each GPS location of the two resolutions with a correlation test (*cor*.*test()* function, “stats” R package). The correlation was > 0.63, we thus kept daily raster maps with the finest resolution for all years (AMSR, res. 6.25 km) except 2012. On that particular year, we rasterized the maps from SSM/I data (res. 12.5 km) on a grid with a resolution of 6.25 km.

Then, sea-ice concentration was averaged over each trip duration (mean of daily sea-ice concentration from the first to the last day of trip) at two spatial-scales (defined below) to have a global view of the overall conditions in the region (scale 1) but also to test the conditions encountered at the trip scale (scale 2):

- The first scale corresponds to the whole study area (Fig 1), between 64° - 67° S and 137° - 143° E, covering 92 770 km^2^ (adapted from [45]). It represents an intermediate area between the maximal extent of foraging trips of Adélie penguins during their incubation and chick-rearing period and encompasses the continental slope and the maximal sea-ice extent recorded in that region over our study period. As such, this scale is representative of the overall conditions that birds can potentially encounter to compare between years.
- The second scale corresponds to the annually prospected area, defined by the annual maximal extent of all penguins’ trips (Fig 1) and is thus more representative of the actual conditions encountered by birds than in the whole study area.

We used the Spearman correlation test to assess if the mean sea-ice conditions in the annually prospected area were representative of the conditions in the whole study area, and if the sea-ice concentrations changed in the same direction using the two scales. We used a Generalized Linear Model (GLM, “stats” R package) to test the effect of the year in interaction with the spatial scale and the timing in the season (the time elapsed between the peak hatching date and the trip departure date) on the mean sea-ice concentration over each trip duration. This helps to compare the inter- and intra-annual variation of the sea-ice scape in the whole study area and to see how it varied around the colony (i.e. in the annually prospected area).

### 2.3. Population-level repeatability in foraging parameters

We measured the repeatability of the four foraging parameters (defined in 2.1) calculated from the entire foraging trip: the total trip duration, the cumulative distance travelled, the maximal distance and the bearing angle between the colony and the most distal point reached during the trip.

To perform repeatability analyses, we first fitted Generalized Linear Mixed Models (GLMMs) with a Gaussian error distribution. All parameters were log-transformed except the bearing angle, to approximate normality of residuals [46]. For each parameter, we fitted a starting model with a two-way interaction between the year and the time elapsed since the peak hatching date as fixed effects, and with bird identity as a random effect. We then selected the best fixed-effects structure, removing non-significant term and comparing models using the AIC. We kept the model with the lowest AIC when the AIC difference was > 2. If the AIC difference was < 2, the most parsimonious model was selected [47]. Models were validated after residuals inspection.

Note that bearing angle was considered as a linear variable, and not as a circular variable, because penguins covered only 44% of the full degree circle (between -71.8° W to 87.8° E).

Using the best GLMM for each parameter (S3.1–S3.4 Tables in S3 Table in S2 File), we calculated the “population-level repeatability” (*R*) with the “rptR” R package using the *rptGaussian()* function [19], as:

$$R=\frac{\sigma_{A}^{2}}{\left(\sigma^{2}+\sigma_{A}^{2}\right)}$$

where $\sigma_{A}^{2}$ is the between-group variance and σ^2^ the global within-group variance. Here, a “group” stands for an individual bird with a set of consecutive trips, so that within and between group variances reflect the variances between trips of a same bird and between individuals, respectively. The repeatability index ranges from 0 to 1: a low repeatability (near 0) reflects either a high within-individual variation or a low between-individual variation. A high repeatability corresponds to a low within-individual variance. Here, we classified the repeatability into three categories (following [8,14]): low (*R* < 0.25), moderate (0.25 > *R* > 0.5) and high (*R* > 0.5). To disentangle what part of the repeatability is explained by external factors (fixed effects) from what is explained by the individual, we compared the adjusted repeatability (i.e. repeatability calculated with the models including both fixed- and random effects, S3.1-S3.4 Tables in S3 Table in S2 File) with the non-adjusted repeatability based on models including only the individual as random effect, without any fixed effect.

### 2.4. Detection of the foraging phase during the trips and repeatability of sea-ice conditions during that phase

In most seabird species including penguins, foraging trips can be divided into an outbound phase to reach suitable foraging grounds, a central phase corresponding to foraging activity, and an inbound phase [2,48–51]. In order to determine the influence of the repeatability of sea-ice conditions on foraging site fidelity, we identified and removed outbound and inbound transit phases to concentrate our analyses on the central foraging phase of the trips of Adélie penguins. To do this, we used the following approach: for each location, we calculated *i)* the distance to the colony (as a straight line) and the percentage of the maximal distance reached during the trip that it represented, and *ii)* the duration elapsed since the departure, as the percentage of the total trip duration. These two variables are related by a bell-shaped curve (S1 Fig in S1 File) on which we calculated two inflection points using the “mcp” R package [52]. The mcp method fits regression models with multiple changing points between generalized linear segments and is based on Bayesian inference. The two inflection points were defined at 31.2% and 69.7% of the trip duration, allowing to identify the three phases of the foraging trips. To further verify that the central phase between the two inflection points concentrated the foraging activity, we calculated the total distance travelled, the mean speed and the mean sinuosity (ratio between the cumulative distance of the segment divided by the straight-line distance between the first and last point of the segment) in each phase for each trip. During the central phase of their trip, birds travelled significantly smaller distances, at slower speeds, and followed more sinuous routes than during the two other phases (S2 Fig in S1 File), which confirmed that foraging activity occurred primarily in the central phase we extracted.

We performed repeatability analysis on the sea-ice conditions encountered during birds’ foraging activity in the central phase of their trip. To do so, we thus calculated the mean sea-ice concentration during the foraging phase by averaging sea-ice values encountered at all GPS locations during that central phase. We then selected the GLMM with the best fixed-effects structure (S3.5 Table in S3 Table in S2 File) and then calculated the adjusted and non-adjusted repeatability, as described in 2.4.

### 2.5. Foraging site fidelity

To assess whether individuals targeted the same foraging areas from one trip to another, we calculated the nearest-neighbour distance (NND), adapted from [53], and following [13] and [54]. This method quantifies the similarity of routes between two trips. Here, we calculated the fidelity from one trip to the next only using the central phase of the trip (see above) and–differently from [53]–only within each individuals, as we only aimed to assess the intra-individual variation in foraging site fidelity. For each combination of successive trips, we extracted the central phase of the first trip (trip _n_) and that of the following trip (trip _n+1_, considered as the focal trip). For each location of the central phase of the trip _n+1_, we calculated the distance to the nearest location of the central phase of the trip _n_. We then calculated the cumulative distance (i.e. the sum of the distances between each location of the trip _n+1_ with the nearest location on the previous trip). Finally, we divided the cumulative distance by the number of locations on the focal trip segment (trip _n+1_), giving a mean distance (the NND) between the two central phases. The greater the fidelity between the foraging areas of two trips, the lower the NND.

We first tested the effect of year on the average annual NND values to assess for inter-annual differences in the foraging site fidelity with a linear model. We then tested the effect of the year in interaction with the timing in the season on the log-transformed NND of the compared trips, using GLMs. We applied a post-hoc test to perform pairwise comparison between years (*emmeans()* function, “emmeans” R package, [55]) and a Tukey adjustment.

We then tested the effect of the mean sea-ice concentration in the annually prospected area over two trips on the NND. We aimed to test if birds returned to close areas–or not–to the location they visited during their previous trip under specific sea-ice conditions. We concomitantly examined variations in sea-ice concentrations between successive trips (difference between the mean sea-ice concentration in the annually prospected area over the first trip and the mean sea-ice concentration in the annually prospected over the next) to account for the potential geographical barriers that sea ice could represent to the movements of the birds from one trip to the next. A positive difference meant that mean sea-ice concentration in the prospected area during the second trip was lower than during the first one (i.e. sea-ice receded), and inversely: a negative sea-ice concentration difference indicates a higher mean sea-ice concentration during the second trip than the previous one. Models were fitted using Generalized Additive Mixed Models (GAMMs, “mgcv” R package) to allow for non-linear relationships. The response variable (NND in the central phase of trip) was log-transformed and bird ID was added as a random effect.

## 3. Results

### 3.1. Foraging activity and sea-ice conditions

Adélie penguins performed longer/farther trips and reached a greater maximal distance in 2011–12 than in other years (Table 2). For all years, birds headed generally North–North-East, and travelled at ca. 3 km.h^-1^ on average, except in 2016–17 where the speed was about 0.5 km.h^-1^.

**Table 2:** Annual mean (± SD) of each foraging parameters: Distances in kilometers, durations in hours, bearing angle between -90° (East) and 90° (West) of the colony.

Year	Total distance (km)	Duration (h)	Maximal distance (km)	Bearing angle (°)
2010–2011	100.38 ± 55.17	34.21 ± 14.87	45.28 ± 24.96	-21.39 ± 21.39
2011–2012	203.85 ± 112.93	76.29 ± 46.98	88.79 ± 50.39	-5.29 ± 21.52
2014–2015	144.17 ± 119.21	59.38 ± 43.87	52.17 ± 29.88	2.10 ± 25.24
2015–2016	43.71 ± 16.14	15.71 ± 4.80	19.71 ± 7.62	-4.45 ± 24.53
2016–2017	16.95 ± 7.31	31.58 ± 4.70	6.25 ± 2.68	-34.13 ± 49.07
2017–2018	127.26 ± 52.67	41.00 ± 16.82	55.11 ± 21.91	-16.25 ± 13.22

The surface of the area prospected by birds differed annually (Fig 1), with the largest area covered by birds’ trips in 2011–12 (23 215.97 km^2^), and the smallest in 2016–17 (740.12 km^2^).

**Fig 2:**
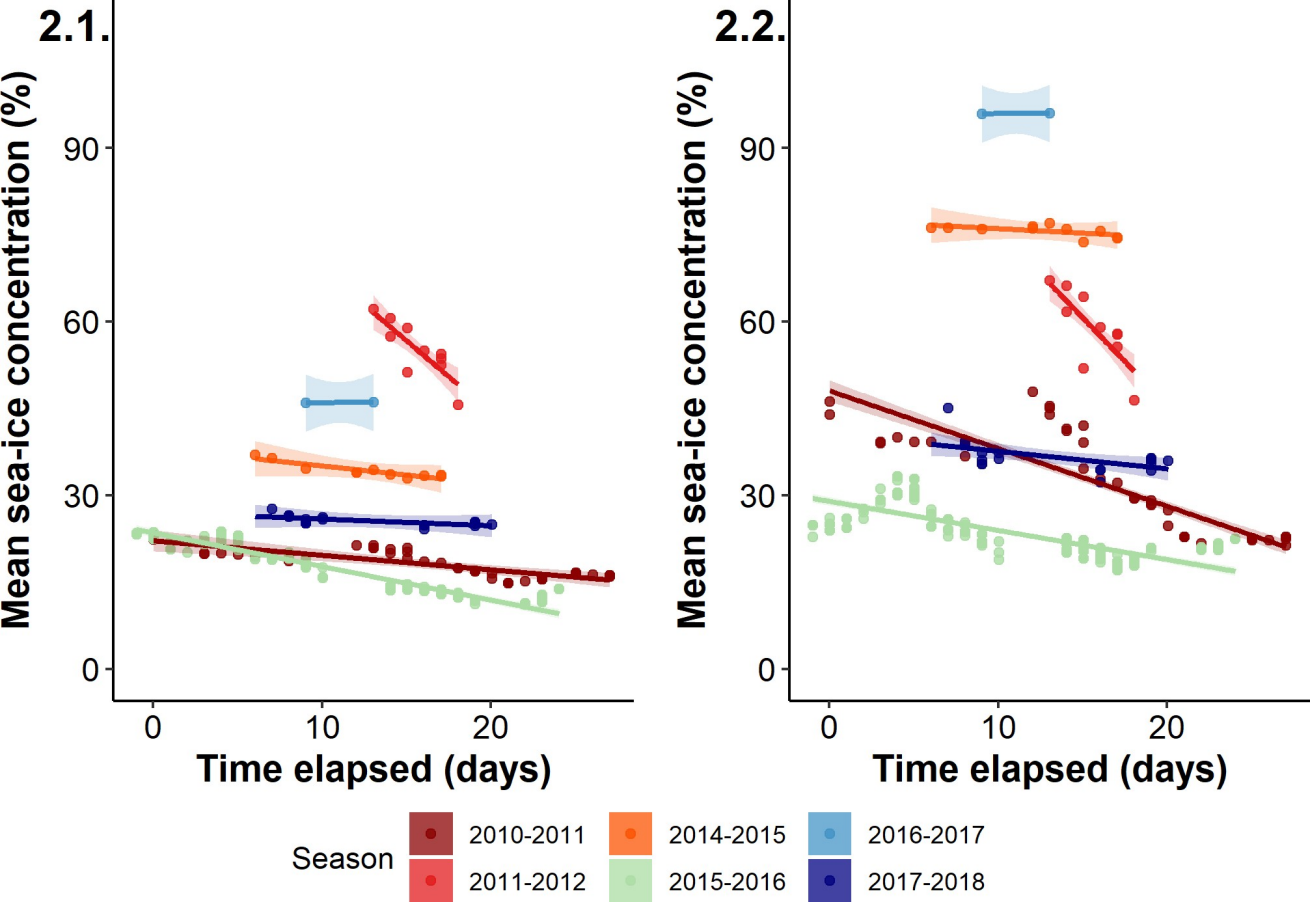
Mean sea-ice concentrations over each trip duration in the whole study area (2.1) and in the annually prospected area (2.2), according to the interaction between the time elapsed since the annual peak hatching date and the year (color code per year).

Mean sea-ice concentration in the whole study area was highly variable among years, except between 2010–11 and 2015–16 which had the lowest values throughout the season (Fig 2.1 in Fig 2, S4 Table in S2 File). Similarly, mean sea-ice concentration in the annually prospected area was highly variable among years (Fig 2.2 in Fig 2, S4 Table in S2 File), although the smallest values were also found in 2015–16.

In addition, within each year, mean sea-ice concentrations over each trip’s combination in the whole study area were correlated to the mean sea-ice concentrations in the annually prospected area (r > 0.75 for all; p < 0.001). At each scale, sea-ice concentrations changed throughout the seasons, but with different directions or amplitudes (Fig 2.1 and 2.2 in Fig 2). In the whole study area (Fig 2.1 in Fig 2), the mean sea-ice concentrations receded significantly throughout the season in all years, except in 2016–17 and 2017–18, with 2011–12 and 2015–16 presenting the most important decrease across the season (S4 Table in S2 File). Considering the annually prospected area, the mean sea-ice concentration did not show any clear tendencies except in 2010–11, 2011–12 and 2015–16 where it receded drastically as the season progressed.

### 3.2.Repeatability in foraging parameters and in environmental conditions

**Fig 3:**
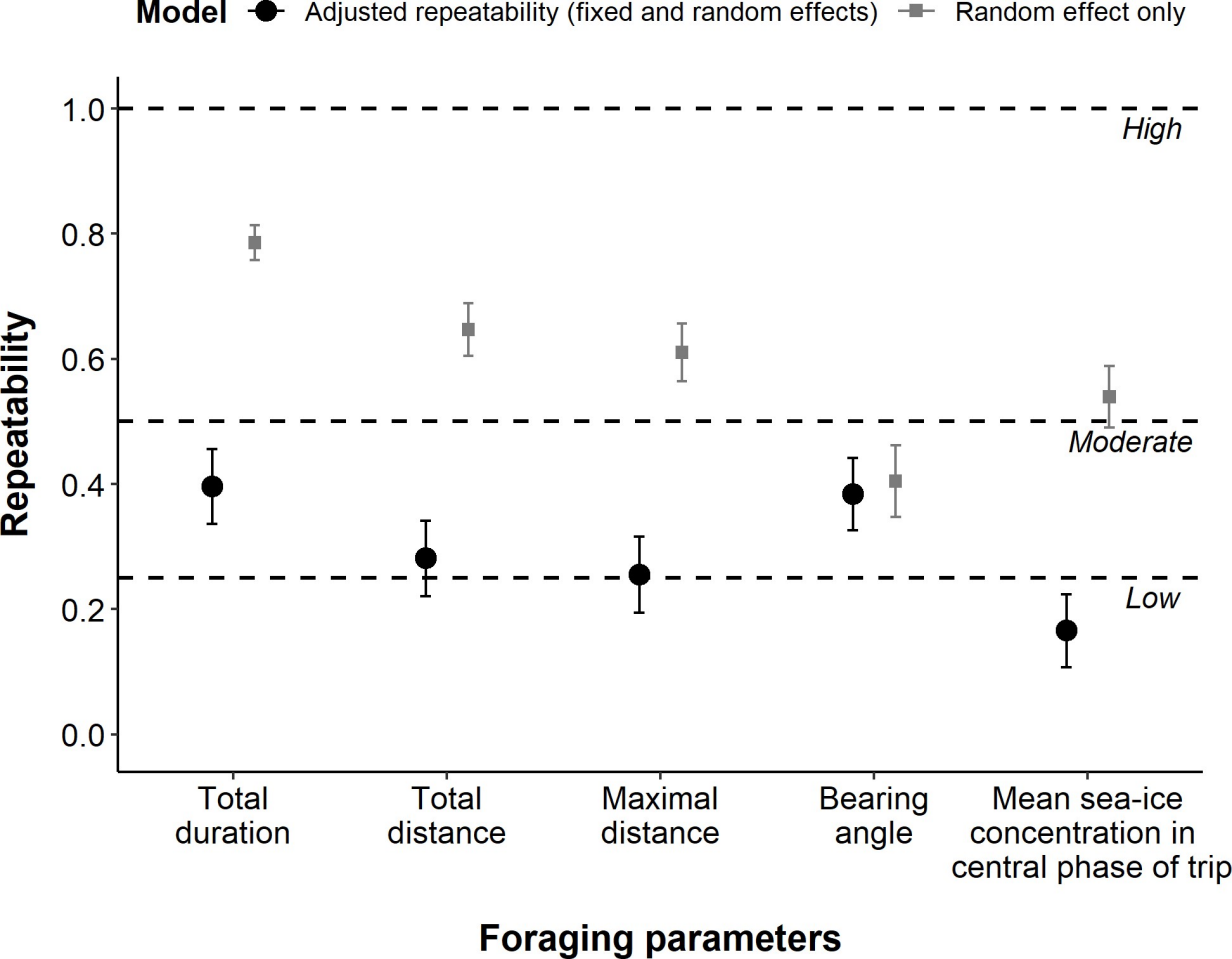
Results of the repeatability (*R* ± SE) analysis on the total trip duration (hours), cumulative distance (km), the maximal distance (km), the bearing angle (transformed between 0 and 180°) between the colony and the most distal point of the trip, and the mean sea-ice concentration in the central phase of trip (%). The large black dots correspond to the results of the adjusted repeatability (models with fixed-effects structure and individual ID as random effect; details of models are given in S3.1-S3.5 Tables in S3 Table in S2 File); the grey squares correspond to the results of the non-adjusted repeatability models using only ID as random effect with no fixed-effect.

Adjusted repeatability (i.e. repeatability calculated with the models including both fixed and random effects, see 2.4) was moderately high for all foraging parameters (*R* ≥ 0.25, Fig 3). Total duration and bearing angle were the highest repeatable parameters (*R* = 0.40 ± 0.06 and 0.38 ± 0.06, respectively) and the maximal distance the least repeatable (*R* = 0.26 ± 0.06). However, during the foraging phase of trips, the sea-ice concentrations encountered by Adélie penguins were poorly repeatable (*R* = 0.17 ± 0.06).

Non-adjusted repeatability values (model including only the ID as random effect and no fixed effect) were higher (*R* > 0.50) for all parameters measured, except for the bearing angle which had approximately the same value as in the adjusted model (*R* = 0.40).

### 3.3.Foraging site fidelity and sea-ice conditions

**Fig 4:**
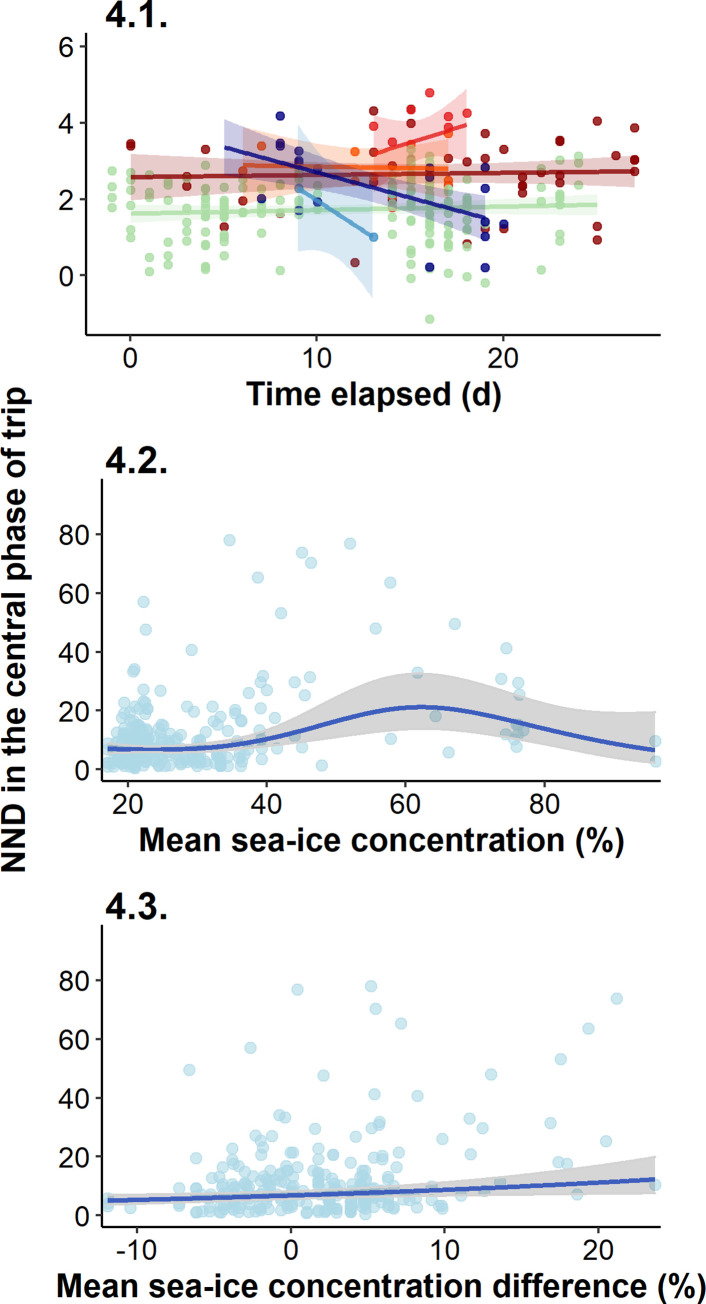
Results of the GLM testing the annual effect of the time elapsed between each trip starting date and the annual peak hatching date (in days) on the NND (log-transformed) in the central phase of trip (mean ± CI, log-scale, Fig 4.1); and GAMM results of the effect of the mean sea-ice concentration on the annually prospected area during two compared trip (%) on the NND in the central phase of trip (back transformed into real scale, Fig 4.2), and effect of the mean sea-ice concentration difference in the annually prospected area between two compared trip (%) on the NND in the central phase of trip (back transformed into real scale, Fig 4.3). On the Fig 4.1, each point corresponds to one trip (see Figs 1 and 2 for annual color code). On the Figs 4.2 and 4.3, one point corresponds to the combination of two trips. A low NND value indicates a high foraging-site fidelity, and inversely a high NND value highlights a low site fidelity.

On average, the foraging site fidelity was the highest in 2015–16 and 2016–17, and the lowest in 2011–12 (Table 3). The site fidelity differed significantly at the beginning of the season. Compared to 2010–11 (the reference), penguins in 2015–16 and 2017–18 exhibited significantly higher and lower site fidelity respectively (Fig 4.1 in Fig 4, S5.1 Table in S5 Table in S2 File), whereas penguins in the other years showed similar site fidelity to that in 2010–11.

**Table 3:** Linear model results of the effect of the year on the NND in the central phase of trip (log-transformed).

Response variable	Predictor variables	Parametric coefficients			p-value
		Estimate	SE	t	
**Log(Trip central phase NND)**	**(Intercept)**	2.667	0.130	20.497	< 0.001
	**2011–2012**	0.914	0.300	3.052	0.003
	**2014–2015**	0.162	0.288	0.561	0.575
	**2015–2016**	-0.946	0.145	-6.516	< 0.001
	**2016–2017**	-1.032	0.617	-1.671	0.096
	**2017–2018**	-0.389	0.235	-1.653	0.100

The change in site fidelity throughout the season differed annually (Fig 4.1 in Fig 4, S5.1 Table in S5 Table in S2 File): it significantly increased throughout the 2017–2018 season but did not change significantly throughout the other seasons, despite slight decreasing trends in 2010–11, 2011–12 and 2015–16, and increasing trends in 2014–15 and 2016–17. The foraging site fidelity in 2011–12 and 2016–17 were more variable compared to other years.

Foraging site fidelity remained high when sea-ice concentration in the annually prospected area averaged between two successive trips ranged from 0 to 30% (Fig 4.2 in Fig 4, S5.2 Table in S5 Table in S2 File). When the mean sea-ice concentration in the area became greater than 30%, foraging site fidelity decreased and showed higher variability, while it increased again beyond 60%. In addition, the foraging site fidelity remained high when sea-ice concentration changed by ± 10% between two trips but decreased and showed greater variability when sea-ice concentration receded by more than 10% from one trip to the next (Fig 4.3 in Fig 4).

## 4. Discussion

While the foraging behaviour of Adélie penguins is repeatable, the repeatability of the environmental conditions in areas prospected by birds is low. In addition, penguins exhibit high foraging site fidelity when the sea-ice concentration around the colony is either low or in greater concentrations, but above all stable. However, under variable sea-ice conditions, penguins increase their exploratory range, as the lowest foraging site fidelity and greater annually prospected areas indicate.

Repeatability has been investigated on a large range of species, from seabirds and raptors to small mammals, using different metrics. It appears to be affected by several factors, including sex, breeding stage, or study sites (see S6 Table in S2 File, S3 Fig in S1 File). Repeatability measured on all foraging parameters of Adélie penguins in our study showed intermediate values, ranging from 0.25 to 0.4 when accounting for the year and the timing in the season in the models. Yet, values were higher for most parameters (except bearing angle) when calculating the repeatability without accounting for year and timing in the season (Fig 3). Such an increase in values between the two models is indicative of low within-individual variation and great between-individuals differences (see methods) across years. In other words, birds within a given year and at the same period in the season adopted similar behaviour as they face the same phenological and environmental constraints. Conversely, repeatability in individuals’ behaviour differs when years and season are not included in the models due to the strong influence of inter-annual variations in environmental conditions.

The adjusted repeatability of Adélie penguins’ trip duration, maximal distance and bearing angle is similar to values measured in another Spheniscidae species, the African penguin (*Spheniscus demersus*, [14]; S3 Fig in S1 File). Interestingly, repeatability in time spent diving–another parameter expressing foraging effort but that we did not measure here–in Adélie penguins from Lützow-Holm Bay was highly variable between years, but also variable between individuals within a breeding season. However, pairs’ consistency was on average moderate within each season ([34]; S6 Table in S2 File, S3 Fig in S1 File). This reflects a possible trade-off between adjusting foraging behaviour to fine-scale changes in their environment and addressing breeding requirements: penguins are time-constrained by their breeding status, but seasonal conditions might influence their foraging behaviour. As offspring demand increases as the season progresses, adults must perform short trips to feed chicks regularly if they are to ensure a good reproductive success, and this must constrain their foraging flexibility. A delay in their return would impact the food delivery to the chick, and ultimately the chick’s survival and/or the partner’s body condition, if this delay was to be repeated. Conversely, shortening too much the trip could lower the body condition of the adult with time, as most of the food collected would be delivered to the chicks and not used for maintaining the body condition of the adults [30,56].

Trip duration and bearing angle were the most repeatable parameters for Adélie penguins in our study. Moreover, repeatability in the bearing angle did not differ between years (no difference between the adjusted and non-adjusted models, Fig 3). This parameter is also the most repeatable in African and gentoo penguins (*Pygoscelis papua*) [57]. Such a consistency in at least three members of the Spheniscidae family reflects the faithfulness to foraging sites, and, accordingly, a potential anticipation of the seasonal spatio-temporal distribution of their prey because of the predictability of resources in given areas, as well as from knowledge accumulated through previous explorations [2,13]. This indicates that Adélie penguins may target previously-known areas where they encountered suitable foraging conditions.

However, the repeatability in sea-ice conditions in the non-adjusted model was higher than that in the adjusted model, reflecting a low intra-individual variability and/or a high inter-individual variability across years. Said differently, penguins faced different conditions among years, but individuals used similar sea-ice conditions under given annual conditions and from one individual trip to another.

Seabirds select their foraging habitats according to prey availability [2,58]. In East Antarctica, during the austral summer, sea-ice conditions are highly variable and penguins should adjust their foraging strategy to face variation in prey distribution [29,59]. The productive foraging grounds are generally found at the sea-ice edge targeted by Adélie penguins during the incubation period [22,24]. When sea-ice retreats over the breeding season, resources become more readily available as foraging grounds become more accessible. During the chick-rearing period, penguins concentrate their efforts on the continental shelf in the absence of sea ice [59] or in polynyas when sea-ice cover remains extensive [27]. Generally, prey patches may follow the recession of sea ice [60,61]. Subsequently, prey depletion can develop around the colony (following the Storer-Ashmole’s theory, [62]). Consistency and site fidelity may thus evolve along with variation in resources distribution and environmental conditions. In other words, the foraging site fidelity may result from a combination of environmental constraints that limit animal movements and decision-making processes where animals try to target (relatively) accessible zones where prey are available.

In our study, while environmental constraints may predominantly explain the high fidelity in foraging areas during years of intensive sea-ice concentration, it is noteworthy that the fidelity is also high in years with no physical constraints to movements. Non-extreme sea-ice conditions, with diffuse sea-ice concentrations around the colony, but enough to enable the resources’ development, offer then the best option for exploration while remaining consistent in respect to phenological time constraint and reproductive effort.

Optimal foraging activity, growth rate and breeding success of Adélie penguins are associated with moderate (up to 20%) sea-ice cover around the colony [33,37,63]. This corresponds well with our findings of a higher foraging site fidelity under such sea-ice concentrations in their prospected area (Fig 4.2 in Fig 4). Adélie penguins forage preferentially in the marginal ice zone, areas of diffuse sea ice where their main prey are abundant [22,28,35,38]. The high fidelity of birds for areas with 0–30% sea-ice concentrations, as well as when the sea-ice coverage varied by ± 10% from one trip to another in the prospected area confirm the preference of Adélie penguins for foraging sites offering easy access to open water near the colony.

When sea-ice concentration is low around the colony from the beginning of the season, penguins have direct access to suitable foraging grounds and exhibit a high site fidelity, like in 2015–16 in our study. However, this takes place in a reduced area of prospection–the smallest after the one prospected in 2016–17 –in which resource availability probably decreased rapidly throughout the season (Fig 1; [62]). Such a decrease may force penguins to increase their prospection range. Yet, they can expect to find new profitable grounds within short distances under low sea-ice concentrations. This is suggested by the high breeding successes observed in the 2010–11 and 2015–16 seasons [30] that are indicative of favourable environmental conditions, and in which there were low sea-ice concentrations, i.e. easy access to productive foraging grounds close to the colony (Figs 1 and 2). The high but constant foraging site fidelity in these years reflects moderate exploration activity across the season when birds track resources which distribution change according to the predatory pressure exerted by birds (Fig 4.1 in Fig 4).

In another case, intermediate but constant sea-ice concentrations throughout the season lead to the adoption of an “exploration-refinement” strategy [13,54], where an increase in foraging site fidelity corresponds to an intermediate breeding success, as in the 2017–18 season (Figs 1, 2 and 4.1 in Fig 4). Under such stable conditions, penguins could explore sites far from the colony to find profitable areas early in the season, but would then reduce their exploration range to concentrate their efforts closer to the colony as the season progresses, consequently reducing the distance between foraging areas. This result might be due to social information transmission about foraging areas to target from one trip to the next, or to the increasing offspring demand forcing penguins to forage closer to ensure rapid and frequent food delivery.

Oppositely, under high sea-ice concentrations, penguins have limited access to open water and must concentrate their foraging effort to the only accessible areas from one trip to the next, as already seen in Lützow-Holm Bay [39]. Yet, prey depletion in these restricted open water areas may be aggravated like in the aforementioned low sea-ice scenario, but here penguins would need to extend their prospective range much farther to encounter new profitable grounds. This was the case in the low breeding success seasons of 2011–12 and 2014–15 (Fig 1; [30]). High sea-ice concentrations from the beginning of the season around the colony (Fig 2) led to low resource availability and rapid prey depletion in close accessible foraging grounds. Birds were then forced to intensify exploration activities farther from the colony. Another extreme example of this is found in the ice-extensive 2016–17 season, with extreme sea-ice concentrations around the colony (Fig 2.2 in Fig 4), where penguins initially fed in ice cracks near the colony (Fig 1; Ropert-Coudert, pers. comm.) but dispersed so far later on the season that they even did not return to their nests [30]. A consequence of this is that only few short trips were available for analysis in that season, leading to a high site fidelity being recorded, in a year where breeding success was null. Yet, as mentioned above, site fidelity under heavy sea-ice concentration reflects physical constraints imposed to penguins that force them to return to the same–and possibly only–areas available. Thus, it may not reflect a deliberate choice of birds to use recurrently resources in the same foraging areas.

Our results concur with others (see [33,35,37,38,63]) to demonstrate that the most suitable conditions for the foraging activities of Adélie penguins consists of easy access to open water but with still diffuse sea-ice conditions favouring, in addition to birds’ movements, the development of resources from different levels of the food chain. In such a setting where multiple sites are potentially profitable, birds can increase their exploratory behaviour to optimize prey access while simultaneously reducing competition for resources.

All our results point out to the highly constrained conditions Adélie penguins face during their breeding season and the impact of the environmental variations on their foraging strategies and consequently on their breeding success. Their contrasted responses to environmental variations highlight the high degree of specialization in resources and environmental conditions Adélie penguins target during foraging (diffuse sea-ice conditions with concentrations up to 30% enabling access to open water near the colony and access to prey). When sea-ice conditions become more variable and resource availability consequently becomes less predictable, birds have to intensify explorative behaviour if they are to optimize prey encounter. Indeed, being flexible may be favourable for individuals to adjust their strategies according to food availability when their habitats undergo fast changes, like is the case in East Antarctica. Bearing in mind the two recent massive breeding failures of the Adélie penguins colony of Pétrels Island that were due to extreme environmental conditions (rain and extreme and persistent sea-ice cover around the colony, see [30,33]), their consistency due to their breeding status prevent them to exhibit more flexibility in their foraging behaviour in order to ensure their breeding success under challenging conditions. Instead, they rather prioritize their own survival abandoning their nest.

## Future directions

Central-place foragers perform their foraging activity around their breeding colony and are thus constrained in the habitat range available in its vicinity [3,64]. There is a growing demonstration that inter-individual variation in foraging behaviour and individual foraging specialization are important component shaping population response to changes occurring in their environment (reviewed in [9,11]). The study of individual specialization and fidelity in foraging site, coupled with the monitoring of the breeding timing, reproductive success and chick quality, could help fully understand how individuals respond to fast environmental changes and how this can shape their reproductive outcome. Moreover, the low flexibility of Adélie penguins in clutch initiation date under increasing air temperature, compared to that of gentoo penguins in the Peninsula [65] also exemplifies the differential response of closely-related species to fast environmental changes. In this context, it would be interesting to determine the levels of behavioural consistency in other species as indices of their plasticity in front of rapid changes in their habitat.

## Supporting information

S1 File (DOCX)

S2 File (DOCX)
