# Supplementary material for: Adélie penguins foraging consistency and site fidelity are conditioned by breeding status and environmental conditions
Source: PLoS One. 2021 Jan 22;16(1):e0244298. doi: 10.1371/journal.pone.0244298 (PMC7822312; doi:10.1371/journal.pone.0244298)
Supplement: S1 File — (DOCX) [file pone.0244298.s001.docx]

**
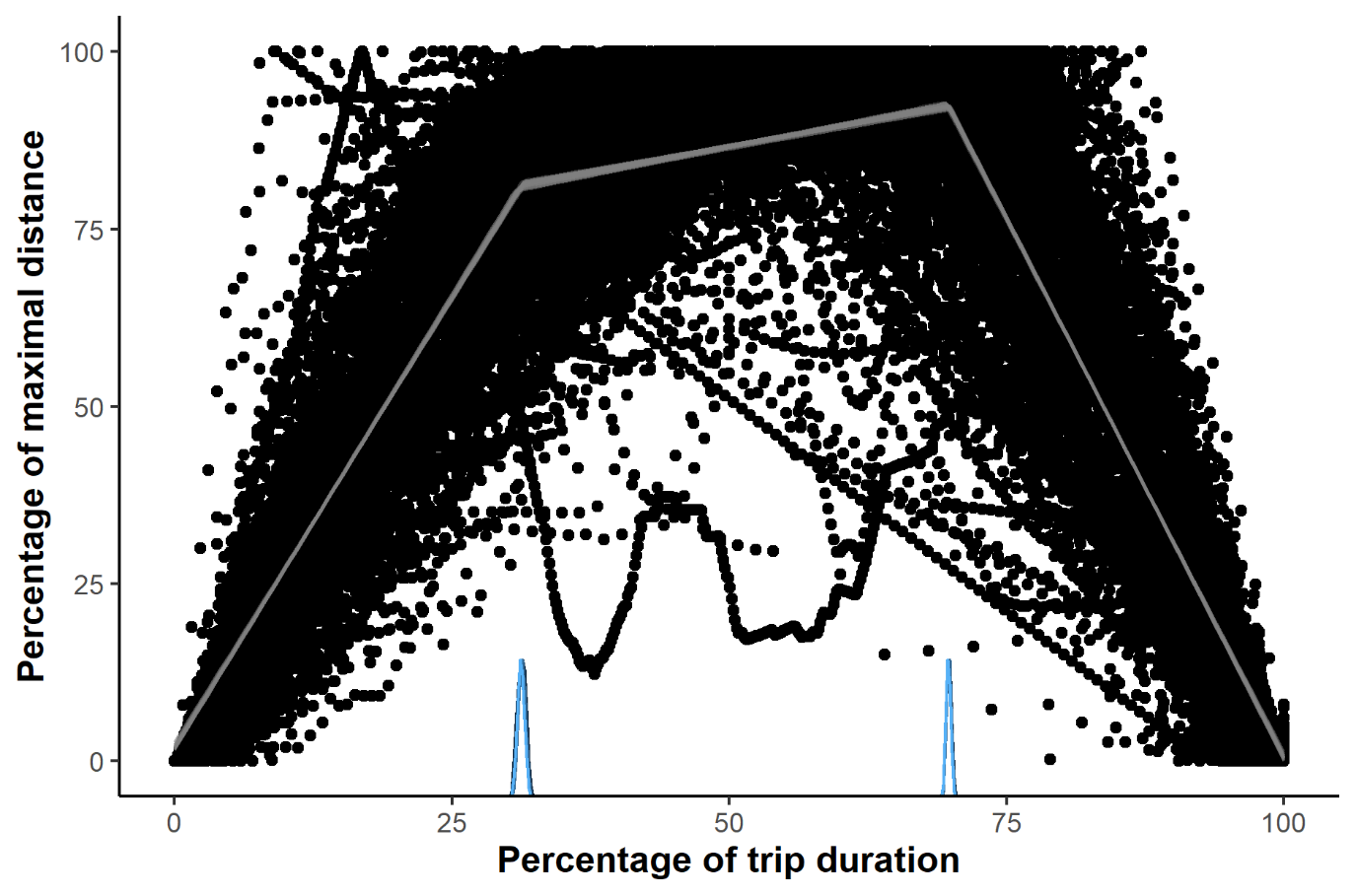
**

**S1 Figure .** Results of the mcp models to find two changing points on the relation between the percentage of the maximal distance (y-axis) according to the percentage of the trip elapsed (x-axis), all locations of all trips confounded. Black points represent each point recorded of all birds’ trips. Grey line represent fitted lines from the regression model, and blue curves the convergence of the models at the given changing points.

**
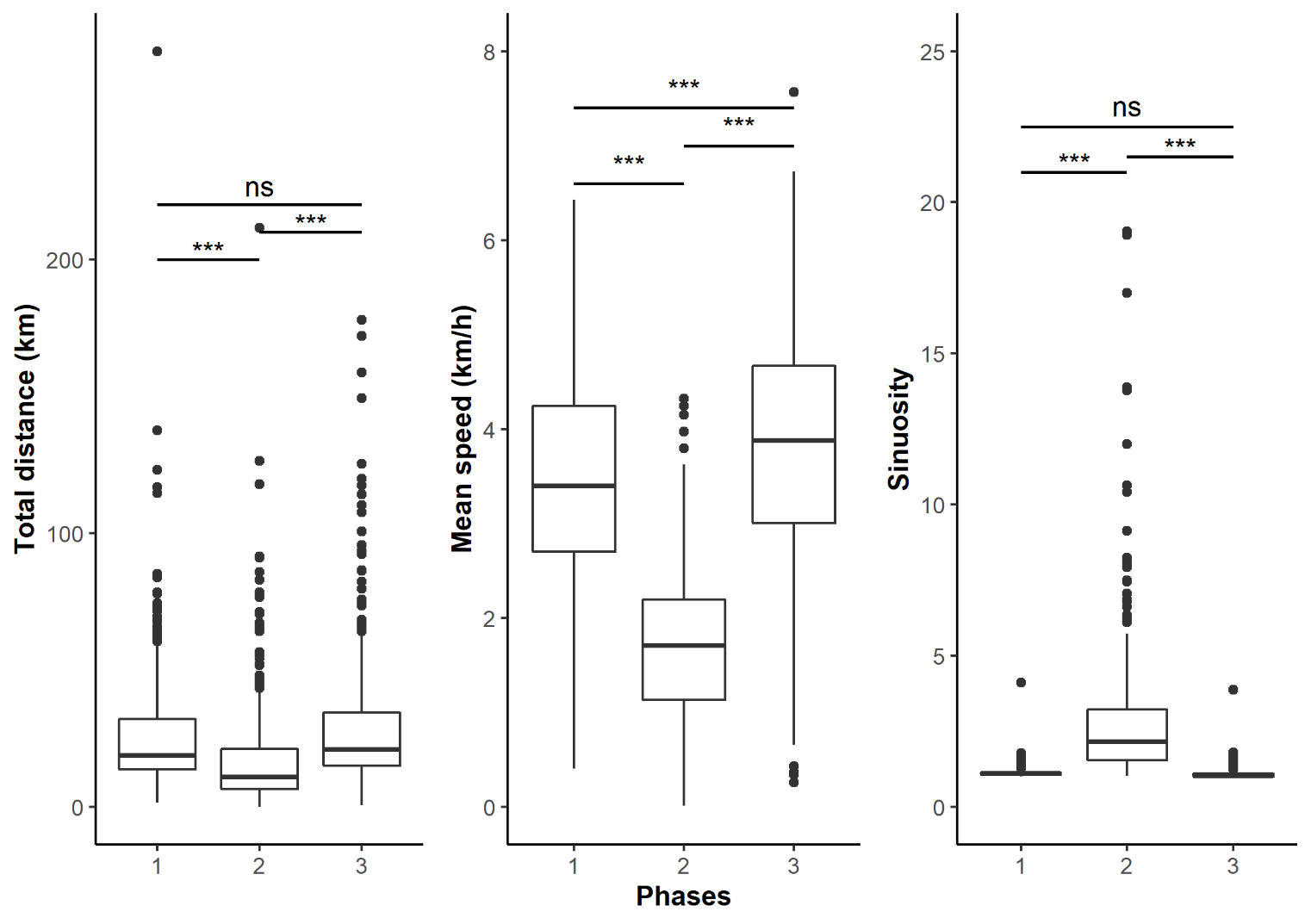
**

**S2 Figure .** Total distance (in kilometres), mean speed (kilometres per hour) and sinuosity calculated on all tracks, on each trip phase based on the two inflections points defined with the mcp method (1 – outbound phase between 0 - 31.2 % of trip duration; 2 - central phase between 31.2 and 69.7 %; and 3 - inbound phase between 69.7 to 100%). Significant differences are indicated as followed: 0.05 > p > 0.01 *; 0.01 > p > 0.001 ** and 0.001 > p ***.


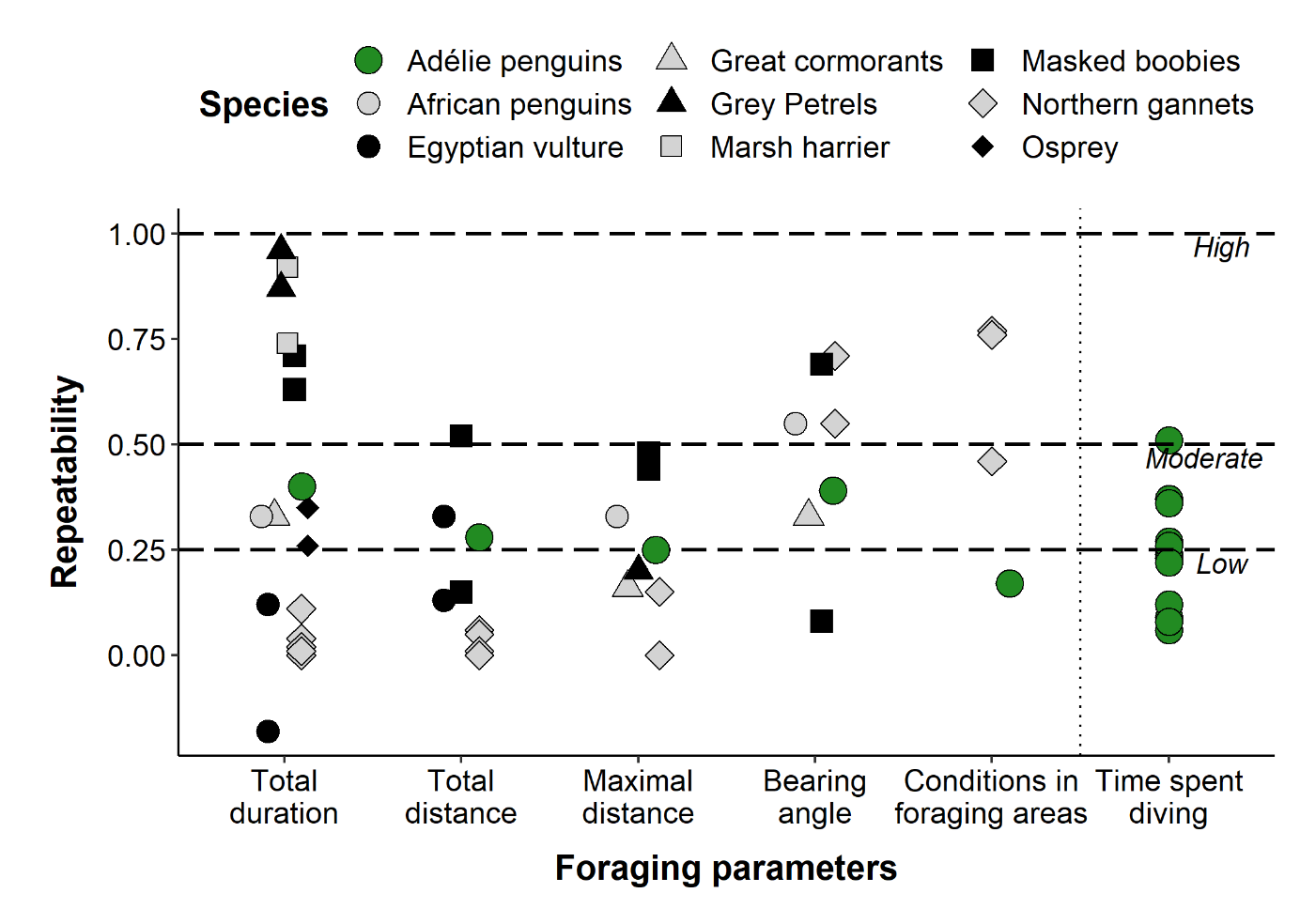


**S3 Figure .** Results of the repeatability (*R*) analysis on the total trip duration, distance, maximal distance, heading between the colony and the most distal point of the trip and the sea-ice concentration average in the trip central phase for Adélie penguins compared to values for the same parameters in other species. The data are from: Traisnel and Pichegru (2019) for the African penguins; López-López et *al*. (2014) for the Egyptian vultures; Potier et *al*. (2015) for the Great cormorants; Delord et *al*. (2019) for the Grey Petrels; Vardanis et *al*. (2016) for the Marsh harriers and Ospreys; Oppel et *al*. (2015) for the Masked boobies; Patrick et *al*. (2014) for the Northern gannets and Takahashi et *al*. (2003) for the data on Adélie penguins time spent diving. Note that for each species, several values for a same parameter are annotated: authors compared repeatability of a parameter on several groups (see Supplementary Table 5 for details and more information on other species for different parameters).
