# Supplementary material for: Adélie penguins foraging consistency and site fidelity are conditioned by breeding status and environmental conditions
Source: PLoS One. 2021 Jan 22;16(1):e0244298. doi: 10.1371/journal.pone.0244298 (PMC7822312; doi:10.1371/journal.pone.0244298)
Supplement: S2 File — (DOCX) [file pone.0244298.s002.docx]

**S1 Table .** Individuals information on foraging trip parameters : bird identity (ID), year of equipment (Year), date-time of first trip departure (“Initial date-time”, UTC+10), date-time of last trip return (“Final date-time”, UTC+10), number of trips recorded (“Nb trips rec.”), trip total distance in km (Mean ± SD), trip duration in hour (Mean ± SD), maximal distance reached (Mean ± SD) and bearing angle between the colony and the most distal point (°, between -90 and 90°).

| **Bird ID** | **Year** | **Initial date-time** | **Final date-time** | **Nb trips rec.** | **Total distance (Mean ± SD)** | | **Duration**  **(Mean ± SD)** | | **Maximal distance (Mean ± SD)** | | **Bearing angle**  **(Mean ± SD)** | |
| --- | --- | --- | --- | --- | --- | --- | --- | --- | --- | --- | --- | --- |
| 2011_66_2 | 2010-2011 | 22/12/2010 23:17 | 31/12/2010 01:34 | 3 | 102.40 | 31.81 | 35.22 | 4.40 | 47.09 | 16.66 | -36.59 | 30.55 |
| 2011_68_2 | 2010-2011 | 22/12/2010 23:45 | 02/01/2011 14:10 | 4 | 96.91 | 58.30 | 28.50 | 6.16 | 45.28 | 28.33 | -4.55 | 9.13 |
| 2011_69_2 | 2010-2011 | 26/12/2010 10:15 | 02/01/2011 10:51 | 2 | 121.50 | 0.47 | 60.17 | 14.38 | 49.29 | 0.79 | 6.79 | 0.04 |
| 2011_73_2 | 2010-2011 | 27/12/2010 20:30 | 31/12/2010 18:38 | 2 | 61.77 | 24.51 | 28.00 | 2.83 | 27.02 | 14.08 | -13.85 | 19.55 |
| 2011_ALPHA | 2010-2011 | 13/01/2011 21:32 | 24/01/2011 11:24 | 5 | 109.89 | 39.16 | 33.80 | 13.29 | 51.02 | 16.78 | -24.51 | 18.76 |
| 2011_F | 2010-2011 | 02/01/2011 07:04 | 03/01/2011 20:36 | 2 | 11.67 | 9.01 | 8.67 | 3.30 | 5.17 | 4.22 | -3.92 | 36.05 |
| 2011_GAMMA | 2010-2011 | 14/01/2011 11:11 | 23/01/2011 11:01 | 4 | 147.62 | 51.62 | 36.08 | 15.31 | 71.92 | 24.51 | -9.22 | 17.85 |
| 2011_J | 2010-2011 | 07/01/2011 14:34 | 15/01/2011 11:06 | 3 | 95.02 | 76.54 | 33.22 | 21.46 | 45.87 | 35.71 | -23.01 | 5.71 |
| 2011_K | 2010-2011 | 07/01/2011 11:23 | 15/01/2011 17:59 | 4 | 104.30 | 79.44 | 34.67 | 22.72 | 49.74 | 37.44 | -10.29 | 12.89 |
| 2011_L | 2010-2011 | 07/01/2011 17:09 | 16/01/2011 07:07 | 3 | 115.20 | 9.59 | 42.44 | 6.43 | 48.87 | 6.44 | -27.80 | 21.22 |
| 2011_P | 2010-2011 | 06/01/2011 17:20 | 16/01/2011 04:27 | 3 | 180.50 | 106.37 | 52.78 | 25.12 | 77.15 | 44.74 | -23.61 | 3.41 |
| 2011_PHI | 2010-2011 | 14/01/2011 11:08 | 24/01/2011 07:24 | 5 | 101.23 | 60.01 | 37.33 | 12.95 | 43.37 | 27.65 | -11.14 | 23.85 |
| 2011_PI | 2010-2011 | 14/01/2011 05:39 | 24/01/2011 15:00 | 5 | 63.76 | 27.79 | 28.40 | 10.30 | 30.05 | 13.23 | -41.62 | 16.52 |
| 2011_Q | 2010-2011 | 05/01/2011 20:42 | 14/01/2011 14:23 | 3 | 123.89 | 29.76 | 33.56 | 4.11 | 49.64 | 15.69 | -3.31 | 10.66 |
| 2011_R | 2010-2011 | 06/01/2011 09:00 | 14/01/2011 01:27 | 3 | 124.52 | 47.53 | 43.67 | 16.65 | 54.98 | 18.57 | -13.75 | 17.89 |
| 2011_SIGMA | 2010-2011 | 14/01/2011 20:29 | 23/01/2011 13:34 | 4 | 63.12 | 28.15 | 29.17 | 11.04 | 28.23 | 11.35 | -44.16 | 9.50 |
| 2011_V | 2010-2011 | 05/01/2011 19:15 | 16/01/2011 13:24 | 5 | 77.19 | 36.91 | 25.00 | 8.13 | 36.05 | 17.91 | -43.64 | 16.88 |
| 2012_BB | 2011-2012 | 08/01/2012 10:13 | 19/01/2012 00:18 | 2 | 246.54 | 15.72 | 101.50 | 6.36 | 92.94 | 9.74 | -15.60 | 2.87 |
| 2012_CC | 2011-2012 | 07/01/2012 18:48 | 19/01/2012 19:57 | 3 | 196.26 | 154.22 | 72.00 | 64.03 | 84.07 | 69.28 | -5.50 | 50.12 |
| 2012_DD | 2011-2012 | 07/01/2012 10:16 | 16/01/2012 10:43 | 2 | 353.81 | 74.68 | 98.50 | 29.46 | 164.52 | 28.27 | 5.55 | 2.32 |
| 2012_EE | 2011-2012 | 08/01/2012 21:16 | 15/01/2012 19:15 | 2 | 191.63 | 44.60 | 58.50 | 22.39 | 87.78 | 15.41 | -6.44 | 5.20 |
| 2012_FF | 2011-2012 | 08/01/2012 09:00 | 18/01/2012 22:37 | 4 | 123.17 | 70.99 | 43.92 | 23.89 | 55.66 | 31.27 | -10.13 | 6.18 |
| 2012_GG | 2011-2012 | 07/01/2012 22:11 | 22/01/2012 06:24 | 2 | 270.80 | 126.06 | 125.50 | 86.50 | 118.62 | 52.30 | -2.11 | 13.56 |
| 2012_JJ | 2011-2012 | 08/01/2012 02:12 | 17/01/2012 04:10 | 2 | 129.22 | 149.81 | 68.67 | 66.47 | 53.45 | 63.06 | 2.17 | 37.04 |
| 2015_B16 | 2014-2015 | 03/01/2015 14:48 | 11/01/2015 11:17 | 2 | 142.21 | 24.92 | 69.17 | 10.14 | 49.35 | 1.51 | 2.80 | 11.89 |
| 2015_V21p | 2014-2015 | 31/12/2014 15:38 | 05/01/2015 13:49 | 2 | 80.23 | 15.59 | 30.83 | 12.02 | 34.99 | 8.40 | -11.02 | 12.26 |
| 2015_V29+ | 2014-2015 | 29/12/2014 14:56 | 02/01/2015 05:23 | 2 | 74.27 | 27.22 | 26.67 | 7.54 | 33.31 | 13.94 | 5.75 | 3.81 |
| 2015_X17 | 2014-2015 | 03/01/2015 04:18 | 08/01/2015 18:24 | 2 | 117.93 | 8.43 | 43.83 | 8.25 | 48.54 | 0.03 | 8.23 | 15.39 |
| 2015_X18 | 2014-2015 | 03/01/2015 04:13 | 09/01/2015 03:51 | 2 | 118.16 | 43.51 | 47.83 | 10.61 | 49.13 | 20.18 | -15.98 | 10.20 |
| 2015_X19 | 2014-2015 | 03/01/2015 17:19 | 09/01/2015 23:44 | 2 | 104.73 | 22.43 | 51.17 | 12.02 | 42.85 | 7.73 | 27.34 | 26.56 |
| 2015_X2 | 2014-2015 | 05/01/2015 12:53 | 12/01/2015 21:25 | 2 | 137.34 | 26.92 | 59.50 | 6.36 | 59.98 | 8.51 | 4.78 | 1.41 |
| 2015_X20 | 2014-2015 | 04/01/2015 12:44 | 15/01/2015 12:54 | 2 | 281.71 | 16.56 | 114.67 | 9.90 | 103.41 | 37.50 | -37.48 | 47.67 |
| 2015_X21 | 2014-2015 | 04/01/2015 11:19 | 18/01/2015 12:01 | 2 | 355.43 | 348.67 | 136.67 | 119.27 | 88.70 | 64.77 | -0.12 | 3.84 |
| 2015_X4 | 2014-2015 | 29/12/2014 22:43 | 03/01/2015 12:31 | 2 | 59.03 | 66.82 | 32.83 | 17.21 | 19.33 | 21.83 | 43.47 | 25.78 |
| 2015_Y2 | 2014-2015 | 06/01/2015 18:01 | 13/01/2015 04:59 | 2 | 114.88 | 11.15 | 40.00 | 8.01 | 44.28 | 0.01 | -4.72 | 4.49 |
| 2016_A1b | 2015-2016 | 08/01/2016 02:23 | 11/01/2016 07:29 | 3 | 46.61 | 11.99 | 15.89 | 1.35 | 21.15 | 5.39 | -6.46 | 5.59 |
| 2016_A2 | 2015-2016 | 27/12/2015 14:46 | 29/12/2015 16:51 | 2 | 36.85 | 13.36 | 13.83 | 2.12 | 16.46 | 6.52 | -12.61 | 13.86 |
| 2016_A2b | 2015-2016 | 22/12/2015 11:02 | 05/01/2016 10:34 | 9 | 48.67 | 12.57 | 22.22 | 2.44 | 21.06 | 6.46 | -16.69 | 23.55 |
| 2016_B1b | 2015-2016 | 07/01/2016 17:12 | 09/01/2016 19:46 | 2 | 60.45 | 0.51 | 15.17 | 0.24 | 27.29 | 3.12 | -29.82 | 14.25 |
| 2016_B2 | 2015-2016 | 27/12/2015 13:17 | 30/12/2015 01:16 | 2 | 26.24 | 12.55 | 12.50 | 2.12 | 11.88 | 6.35 | 4.41 | 15.94 |
| 2016_B2b | 2015-2016 | 22/12/2015 08:38 | 04/01/2016 17:58 | 8 | 35.55 | 14.50 | 15.08 | 3.17 | 16.24 | 6.78 | 0.29 | 23.27 |
| 2016_Bb | 2015-2016 | 06/01/2016 21:34 | 10/01/2016 07:01 | 3 | 56.16 | 9.51 | 15.56 | 0.77 | 26.35 | 4.13 | -18.46 | 20.24 |
| 2016_C1b | 2015-2016 | 08/01/2016 01:43 | 13/01/2016 02:26 | 5 | 40.70 | 15.11 | 12.47 | 2.96 | 19.30 | 7.55 | -1.85 | 14.91 |
| 2016_C2 | 2015-2016 | 28/12/2015 01:39 | 30/12/2015 05:19 | 2 | 41.63 | 4.47 | 15.17 | 0.24 | 16.78 | 3.67 | -0.31 | 7.09 |
| 2016_C2b | 2015-2016 | 24/12/2015 02:08 | 04/01/2016 02:22 | 6 | 41.57 | 7.23 | 20.39 | 1.44 | 16.88 | 3.19 | 31.90 | 11.62 |
| 2016_C3b | 2015-2016 | 11/01/2016 09:17 | 14/01/2016 07:37 | 3 | 47.00 | 6.74 | 13.56 | 1.17 | 21.12 | 2.85 | 4.81 | 25.94 |
| 2016_Cb | 2015-2016 | 07/01/2016 04:11 | 13/01/2016 06:52 | 5 | 38.29 | 10.85 | 14.53 | 2.69 | 17.52 | 5.69 | 13.95 | 11.43 |
| 2016_D1b | 2015-2016 | 08/01/2016 01:42 | 10/01/2016 09:15 | 2 | 65.68 | 2.26 | 15.50 | 0.24 | 31.43 | 1.24 | -19.87 | 26.98 |
| 2016_D2 | 2015-2016 | 28/12/2015 02:50 | 30/12/2015 16:20 | 2 | 43.73 | 3.69 | 18.33 | 0.94 | 20.08 | 2.73 | -16.15 | 4.10 |
| 2016_D2b | 2015-2016 | 22/12/2015 17:45 | 04/01/2016 20:20 | 9 | 44.08 | 9.89 | 16.59 | 2.67 | 20.42 | 5.06 | -24.43 | 21.16 |
| 2016_D3b | 2015-2016 | 10/01/2016 18:11 | 12/01/2016 13:27 | 2 | 42.75 | 9.39 | 12.83 | 2.59 | 20.65 | 4.37 | -30.57 | 8.00 |
| 2016_Db | 2015-2016 | 06/01/2016 16:30 | 11/01/2016 13:31 | 5 | 43.88 | 10.69 | 11.53 | 1.48 | 20.37 | 4.70 | -17.86 | 14.97 |
| 2016_E1b | 2015-2016 | 07/01/2016 20:30 | 10/01/2016 15:59 | 3 | 40.19 | 11.16 | 12.44 | 3.89 | 18.41 | 5.95 | 8.79 | 27.62 |
| 2016_E2 | 2015-2016 | 28/12/2015 05:17 | 30/12/2015 21:43 | 2 | 35.97 | 4.63 | 14.50 | 0.71 | 16.01 | 2.60 | -14.87 | 13.02 |
| 2016_E2b | 2015-2016 | 23/12/2015 15:12 | 03/01/2016 09:32 | 7 | 42.13 | 11.89 | 15.81 | 2.63 | 19.45 | 5.29 | -11.66 | 18.39 |
| 2016_E3b | 2015-2016 | 15/01/2016 02:16 | 17/01/2016 10:41 | 2 | 52.93 | 16.62 | 18.00 | 1.89 | 23.08 | 8.55 | -7.84 | 25.70 |
| 2016_F1b | 2015-2016 | 08/01/2016 11:46 | 10/01/2016 14:17 | 2 | 42.06 | 17.36 | 14.67 | 3.77 | 20.00 | 8.24 | 6.23 | 13.85 |
| 2016_F2 | 2015-2016 | 28/12/2015 05:43 | 30/12/2015 17:46 | 2 | 28.97 | 8.36 | 13.50 | 1.65 | 13.16 | 3.62 | -16.39 | 14.32 |
| 2016_F2b | 2015-2016 | 23/12/2015 06:21 | 04/01/2016 09:19 | 9 | 33.58 | 3.52 | 15.93 | 2.70 | 15.33 | 2.13 | 6.42 | 17.25 |
| 2016_F3b | 2015-2016 | 14/01/2016 23:31 | 18/01/2016 01:10 | 3 | 53.24 | 2.36 | 15.56 | 1.84 | 24.37 | 2.16 | -10.55 | 6.16 |
| 2016_Fb | 2015-2016 | 06/01/2016 22:14 | 11/01/2016 17:54 | 5 | 28.81 | 16.80 | 10.60 | 2.62 | 13.05 | 7.84 | 23.02 | 21.46 |
| 2016_G1b | 2015-2016 | 09/01/2016 03:09 | 13/01/2016 09:08 | 4 | 22.55 | 15.57 | 15.17 | 4.34 | 10.26 | 7.64 | 24.32 | 10.40 |
| 2016_G2b | 2015-2016 | 22/12/2015 22:49 | 04/01/2016 19:24 | 7 | 70.83 | 19.70 | 26.95 | 5.98 | 29.73 | 8.75 | -12.20 | 26.80 |
| 2016_Gb | 2015-2016 | 07/01/2016 02:42 | 11/01/2016 18:07 | 4 | 53.96 | 10.50 | 15.83 | 3.36 | 24.17 | 6.37 | -12.03 | 7.28 |
| 2016_H1b | 2015-2016 | 08/01/2016 12:33 | 14/01/2016 00:49 | 5 | 46.81 | 12.84 | 15.27 | 2.80 | 20.89 | 6.75 | -0.16 | 18.78 |
| 2016_H2b | 2015-2016 | 23/12/2015 08:09 | 04/01/2016 08:34 | 8 | 40.99 | 12.53 | 13.21 | 2.54 | 19.55 | 6.28 | -5.15 | 14.39 |
| 2016_H3b | 2015-2016 | 15/01/2016 12:45 | 17/01/2016 18:49 | 2 | 48.93 | 6.23 | 17.67 | 4.24 | 21.36 | 1.89 | -30.11 | 33.74 |
| 2016_I2b | 2015-2016 | 23/12/2015 02:32 | 29/12/2015 23:47 | 4 | 48.68 | 9.30 | 23.08 | 2.42 | 19.61 | 3.72 | -15.58 | 24.84 |
| 2016_I3b | 2015-2016 | 15/01/2016 17:45 | 17/01/2016 14:59 | 2 | 53.80 | 16.76 | 16.83 | 2.59 | 23.36 | 8.45 | -19.13 | 20.26 |
| 2016_Ib | 2015-2016 | 06/01/2016 15:23 | 14/01/2016 08:18 | 5 | 50.81 | 22.88 | 21.40 | 12.92 | 22.97 | 11.27 | 17.42 | 31.33 |
| 2016_J2 | 2015-2016 | 28/12/2015 03:50 | 30/12/2015 10:53 | 2 | 39.33 | 0.57 | 16.83 | 1.18 | 17.29 | 1.47 | -9.24 | 3.49 |
| 2016_J2b | 2015-2016 | 23/12/2015 11:46 | 04/01/2016 04:08 | 8 | 37.58 | 15.24 | 16.00 | 5.68 | 16.93 | 6.87 | 1.14 | 23.14 |
| 2016_Jb | 2015-2016 | 07/01/2016 01:50 | 10/01/2016 13:52 | 3 | 43.23 | 21.15 | 16.22 | 3.75 | 19.80 | 10.86 | 1.46 | 8.56 |
| 2016_K2 | 2015-2016 | 28/12/2015 05:48 | 30/12/2015 12:27 | 2 | 28.91 | 6.89 | 13.50 | 2.12 | 12.54 | 2.31 | -5.56 | 27.80 |
| 2016_K3b | 2015-2016 | 15/01/2016 19:29 | 18/01/2016 07:03 | 2 | 70.04 | 24.28 | 19.67 | 0.94 | 32.11 | 11.71 | -51.33 | 18.50 |
| 2016_Kb | 2015-2016 | 07/01/2016 07:00 | 10/01/2016 14:56 | 3 | 43.19 | 17.90 | 12.44 | 2.14 | 20.91 | 8.53 | 0.43 | 9.06 |
| 2016_L1b | 2015-2016 | 08/01/2016 16:18 | 10/01/2016 09:09 | 2 | 48.72 | 0.23 | 11.83 | 0.24 | 24.06 | 0.05 | -14.48 | 12.78 |
| 2016_L2 | 2015-2016 | 27/12/2015 23:44 | 30/12/2015 18:36 | 2 | 45.41 | 7.05 | 20.00 | 1.89 | 18.28 | 0.73 | -23.09 | 2.68 |
| 2016_L3b | 2015-2016 | 15/01/2016 22:09 | 17/01/2016 18:40 | 2 | 29.28 | 12.30 | 18.50 | 10.61 | 13.85 | 5.14 | 1.73 | 23.78 |
| 2016_Lb | 2015-2016 | 06/01/2016 20:14 | 12/01/2016 07:36 | 5 | 54.53 | 16.02 | 13.33 | 2.53 | 25.37 | 7.19 | -30.08 | 9.12 |
| 2016_M2 | 2015-2016 | 28/12/2015 01:46 | 30/12/2015 05:32 | 2 | 38.76 | 7.29 | 15.00 | 0.00 | 17.01 | 4.40 | 3.29 | 1.18 |
| 2016_M3b | 2015-2016 | 16/01/2016 19:33 | 18/01/2016 18:22 | 2 | 60.98 | 23.29 | 15.50 | 0.24 | 27.55 | 12.46 | -5.28 | 30.30 |
| 2016_N1b | 2015-2016 | 08/01/2016 18:57 | 13/01/2016 03:40 | 4 | 36.26 | 12.68 | 14.00 | 3.17 | 16.03 | 6.50 | -36.04 | 14.90 |
| 2016_N2 | 2015-2016 | 28/12/2015 09:03 | 30/12/2015 20:29 | 2 | 29.03 | 4.43 | 13.83 | 1.18 | 12.72 | 2.49 | 0.57 | 20.14 |
| 2016_N3b | 2015-2016 | 15/01/2016 22:27 | 17/01/2016 19:42 | 3 | 34.60 | 9.69 | 8.89 | 1.35 | 16.40 | 4.52 | 11.13 | 6.65 |
| 2016_Nb | 2015-2016 | 07/01/2016 11:37 | 09/01/2016 13:18 | 2 | 54.16 | 38.93 | 15.33 | 4.24 | 23.63 | 19.77 | 15.81 | 25.27 |
| 2016_O1b | 2015-2016 | 09/01/2016 02:28 | 14/01/2016 09:43 | 4 | 44.58 | 3.46 | 16.75 | 1.26 | 19.18 | 2.33 | -1.18 | 6.21 |
| 2016_O3b | 2015-2016 | 16/01/2016 03:08 | 17/01/2016 19:02 | 2 | 36.70 | 6.57 | 10.83 | 1.65 | 17.83 | 3.31 | -1.93 | 5.44 |
| 2016_Ob | 2015-2016 | 07/01/2016 18:34 | 11/01/2016 16:43 | 4 | 17.17 | 4.32 | 9.58 | 1.20 | 7.13 | 1.89 | 64.81 | 16.56 |
| 2016_Pb | 2015-2016 | 07/01/2016 16:41 | 11/01/2016 08:13 | 3 | 53.05 | 19.88 | 17.89 | 1.39 | 24.72 | 11.06 | 10.39 | 20.64 |
| 2016_Q1b | 2015-2016 | 08/01/2016 22:34 | 12/01/2016 11:54 | 3 | 53.36 | 16.72 | 21.56 | 5.39 | 23.51 | 9.57 | 6.32 | 21.64 |
| 2016_Q2 | 2015-2016 | 28/12/2015 08:29 | 30/12/2015 15:58 | 2 | 55.84 | 25.45 | 17.50 | 5.89 | 23.15 | 11.46 | -9.51 | 31.14 |
| 2016_Qb | 2015-2016 | 07/01/2016 21:06 | 12/01/2016 06:24 | 3 | 61.40 | 19.97 | 20.56 | 4.44 | 27.72 | 9.96 | -29.11 | 16.28 |
| 2016_R1b | 2015-2016 | 09/01/2016 11:57 | 11/01/2016 12:40 | 2 | 48.74 | 5.75 | 16.33 | 0.47 | 20.11 | 3.70 | -1.51 | 10.85 |
| 2016_Rb | 2015-2016 | 08/01/2016 01:23 | 09/01/2016 21:44 | 2 | 61.50 | 9.12 | 14.50 | 1.18 | 29.93 | 5.60 | -7.44 | 11.30 |
| 2016_S1b | 2015-2016 | 09/01/2016 11:29 | 12/01/2016 17:02 | 3 | 46.52 | 5.56 | 15.56 | 1.35 | 21.89 | 3.26 | 13.79 | 18.01 |
| 2016_S2 | 2015-2016 | 28/12/2015 17:17 | 31/12/2015 03:28 | 2 | 32.92 | 3.35 | 14.00 | 3.77 | 13.55 | 1.88 | -22.67 | 15.73 |
| 2016_S3b | 2015-2016 | 16/01/2016 16:30 | 18/01/2016 07:08 | 2 | 30.81 | 34.20 | 11.67 | 3.77 | 14.47 | 15.80 | -12.75 | 10.25 |
| 2016_Sb | 2015-2016 | 08/01/2016 07:10 | 11/01/2016 14:14 | 3 | 56.28 | 16.24 | 13.44 | 2.87 | 26.72 | 7.98 | -35.37 | 20.60 |
| 2016_T1b | 2015-2016 | 10/01/2016 20:24 | 12/01/2016 13:32 | 2 | 33.77 | 2.81 | 11.50 | 0.24 | 15.52 | 1.07 | 5.76 | 17.06 |
| 2016_T3b | 2015-2016 | 16/01/2016 22:07 | 18/01/2016 18:53 | 2 | 52.16 | 39.71 | 13.83 | 5.89 | 23.58 | 16.15 | -31.20 | 19.98 |
| 2016_Tb | 2015-2016 | 08/01/2016 01:09 | 10/01/2016 14:33 | 3 | 40.82 | 23.18 | 12.00 | 3.28 | 18.86 | 11.35 | 21.74 | 23.60 |
| 2016_U1b | 2015-2016 | 10/01/2016 11:12 | 12/01/2016 06:34 | 2 | 43.50 | 2.25 | 12.83 | 1.65 | 19.68 | 2.35 | -37.70 | 5.47 |
| 2016_U3b | 2015-2016 | 16/01/2016 22:15 | 18/01/2016 21:31 | 2 | 58.56 | 14.74 | 15.17 | 2.59 | 28.05 | 7.76 | -11.91 | 19.67 |
| 2016_Ub | 2015-2016 | 08/01/2016 05:47 | 10/01/2016 16:42 | 2 | 54.46 | 34.01 | 16.83 | 7.78 | 25.68 | 17.64 | 3.68 | 21.04 |
| 2016_Vb | 2015-2016 | 08/01/2016 01:25 | 12/01/2016 13:52 | 4 | 34.72 | 18.82 | 12.75 | 2.82 | 15.12 | 9.59 | 17.34 | 14.81 |
| 2016_X2 | 2015-2016 | 28/12/2015 12:10 | 30/12/2015 11:55 | 2 | 32.91 | 0.81 | 12.17 | 0.24 | 13.82 | 0.92 | -21.60 | 4.13 |
| 2016_Z2 | 2015-2016 | 29/12/2015 00:23 | 30/12/2015 13:42 | 2 | 19.28 | 3.66 | 8.67 | 2.83 | 9.01 | 1.63 | -31.70 | 2.88 |
| 2017_C15 | 2016-2017 | 30/12/2016 06:21 | 03/01/2017 09:11 | 2 | 21.86 | 2.23 | 33.17 | 5.42 | 7.07 | 2.91 | -11.25 | 71.54 |
| 2017_K3 | 2016-2017 | 02/01/2017 14:17 | 06/01/2017 22:10 | 2 | 12.04 | 7.68 | 30.00 | 5.19 | 5.42 | 3.22 | -57.02 | 3.15 |
| 2018_C13 | 2017-2018 | 26/12/2017 19:48 | 31/12/2017 21:07 | 2 | 139.66 | 68.42 | 39.00 | 14.61 | 65.27 | 30.38 | -18.31 | 1.10 |
| 2018_C15 | 2017-2018 | 28/12/2017 00:10 | 01/01/2018 15:37 | 2 | 91.34 | 26.72 | 30.50 | 6.36 | 36.57 | 5.99 | 1.36 | 0.52 |
| 2018_C16 | 2017-2018 | 27/12/2017 13:31 | 01/01/2018 13:03 | 2 | 99.64 | 11.37 | 40.67 | 2.83 | 42.74 | 4.90 | -11.79 | 2.25 |
| 2018_C17 | 2017-2018 | 27/12/2017 11:37 | 01/01/2018 00:28 | 2 | 93.41 | 34.70 | 32.83 | 4.48 | 41.57 | 17.20 | -19.73 | 3.59 |
| 2018_C18 | 2017-2018 | 27/12/2017 16:21 | 01/01/2018 15:32 | 2 | 99.40 | 9.31 | 36.50 | 7.31 | 40.15 | 4.44 | -25.63 | 23.15 |
| 2018_C2 | 2017-2018 | 24/12/2017 20:16 | 29/12/2017 13:57 | 2 | 124.29 | 17.57 | 35.17 | 1.65 | 54.98 | 4.17 | -43.40 | 1.75 |
| 2018_C22 | 2017-2018 | 27/12/2017 17:59 | 02/01/2018 12:14 | 2 | 164.30 | 35.93 | 45.33 | 4.71 | 73.20 | 10.93 | -1.96 | 1.83 |
| 2018_C32 | 2017-2018 | 03/01/2018 00:57 | 07/01/2018 13:50 | 2 | 130.26 | 39.38 | 39.50 | 12.96 | 55.33 | 16.88 | 3.22 | 0.21 |
| 2018_C34 | 2017-2018 | 03/01/2018 17:31 | 07/01/2018 17:56 | 2 | 95.33 | 2.52 | 32.83 | 3.54 | 42.74 | 2.45 | -11.48 | 0.33 |
| 2018_C40 | 2017-2018 | 04/01/2018 04:07 | 08/01/2018 11:36 | 2 | 125.70 | 4.08 | 40.17 | 3.54 | 52.47 | 3.96 | -8.19 | 13.71 |
| 2018_C5 | 2017-2018 | 26/12/2017 13:22 | 31/12/2017 11:35 | 2 | 94.55 | 47.12 | 33.50 | 11.55 | 40.17 | 25.60 | -30.23 | 14.64 |
| 2018_C53 | 2017-2018 | 05/01/2018 19:13 | 11/01/2018 21:48 | 2 | 179.78 | 11.45 | 65.33 | 16.03 | 80.50 | 4.10 | -32.45 | 6.66 |
| 2018_C54 | 2017-2018 | 07/01/2018 04:08 | 11/01/2018 03:59 | 2 | 93.79 | 2.55 | 31.00 | 1.89 | 42.01 | 1.41 | -7.77 | 5.33 |
| 2018_C57 | 2017-2018 | 06/01/2018 16:33 | 11/01/2018 08:31 | 2 | 136.91 | 31.16 | 39.17 | 10.14 | 62.96 | 13.72 | -24.75 | 5.27 |
| 2018_C58 | 2017-2018 | 06/01/2018 17:05 | 10/01/2018 20:25 | 2 | 103.71 | 6.94 | 30.00 | 1.89 | 46.60 | 2.60 | -21.16 | 5.87 |
| 2018_C59 | 2017-2018 | 06/01/2018 17:45 | 11/01/2018 10:19 | 2 | 118.51 | 2.36 | 41.00 | 2.36 | 49.36 | 1.66 | -22.11 | 1.38 |
| 2018_C6 | 2017-2018 | 26/12/2017 15:07 | 01/01/2018 19:41 | 2 | 212.85 | 66.38 | 59.83 | 11.08 | 92.44 | 27.02 | -13.71 | 5.24 |
| 2018_C60 | 2017-2018 | 08/01/2018 04:59 | 11/01/2018 12:42 | 2 | 95.17 | 4.48 | 26.17 | 2.12 | 43.78 | 2.65 | -12.07 | 9.41 |
| 2018_C7 | 2017-2018 | 26/12/2017 17:24 | 03/01/2018 18:15 | 2 | 219.27 | 163.61 | 80.50 | 51.15 | 84.24 | 65.33 | -8.62 | 9.11 |

|  |  |  |  |  |  |  |  |  |  |  |  |  |
| --- | --- | --- | --- | --- | --- | --- | --- | --- | --- | --- | --- | --- |
|  |  |  |  |  |  |  |  |  |  |  |  |  |

**S2 Table .** Annual number of nests in our monitored colony for which at least the first egg successfully hatched, with the date of the annual peak date calculated as the median date of the first egg hatching.

| **Year** | **Number of nests** | **Peak hatching date** |
| --- | --- | --- |
| **2010-2011** | 44 | 26-Dec-2010 |
| **2011-2012** | 30 | 2-Dec-2012 |
| **2014-2015** | 73 | 25-Dec-2014 |
| **2015-2016** | 79 | 25-Dec-2015 |
| **2016-2017** | 51 | 23-Dec-2016 |
| **2017-2018** | 71 | 21-Dec-2017 |

**S3 Tables .** GLMMs results after model selection for foraging parameters (Duration, S3.1 Table; Distance, S3.2 Table; Maximal distance, S3.3 Table; Bearing angle S3.4 Table) and sea-ice conditions in the central phase of trip (S3.5 Table).

- **S3.1 Table : Duration model .** Effect of the year and the year in interaction with the timing in the season on the trip duration (log-transformed).

***Model : log(Total duration) ~ Year + Year : Time elapsed since the hatching peak date***

|  | **Value** | **Std.Error** | **DF** | **t-value** | **p-value** |
| --- | --- | --- | --- | --- | --- |
| **(Intercept)** | 3.163 | 0.117 | 254.000 | 27.078 | < 0.001 |
| **2011-2012** | -0.333 | 0.375 | 123.000 | -0.889 | 0.376 |
| **2014-2015** | -0.222 | 0.256 | 123.000 | -0.867 | 0.387 |
| **2015-2016** | -0.340 | 0.131 | 123.000 | -2.596 | 0.011 |
| **2016-2017** | -0.090 | 0.822 | 123.000 | -0.110 | 0.913 |
| **2017-2018** | 0.519 | 0.197 | 123.000 | 2.630 | 0.010 |
| **2010-2011:Time elapsed** | 0.019 | 0.007 | 254.000 | 2.811 | 0.005 |
| **2011-2012:Time elapsed** | 0.101 | 0.025 | 254.000 | 4.003 | < 0.001 |
| **2014-2015:Time elapsed** | 0.092 | 0.020 | 254.000 | 4.628 | < 0.001 |
| **2015-2016:Time elapsed** | -0.011 | 0.004 | 254.000 | -2.635 | 0.009 |
| **2016-2017:Time elapsed** | 0.038 | 0.080 | 254.000 | 0.473 | 0.637 |
| **2017-2018:Time elapsed** | -0.002 | 0.012 | 254.000 | -0.177 | 0.860 |

- **S3.2 Table : Distance model .** Effect of the year in interaction with the timing in the season on the trip total distance (log-transformed).

***Model : log(Total distance) ~ Year * Time elapsed since the hatching peak date***

|  | **Value** | **Std.Error** | **DF** | **t-value** | **p-value** |
| --- | --- | --- | --- | --- | --- |
| **(Intercept)** | 4.037 | 0.160 | 254.000 | 25.304 | < 0.001 |
| **2011-2012** | 0.371 | 0.563 | 123.000 | 0.659 | 0.511 |
| **2014-2015** | -0.602 | 0.363 | 123.000 | -1.657 | 0.100 |
| **2015-2016** | -0.345 | 0.178 | 123.000 | -1.935 | 0.055 |
| **2016-2017** | -1.464 | 1.185 | 123.000 | -1.236 | 0.219 |
| **2017-2018** | 0.669 | 0.271 | 123.000 | 2.467 | 0.015 |
| **Time elapsed** | 0.026 | 0.010 | 254.000 | 2.780 | 0.006 |
| **2011-2012:Time elapsed** | 0.027 | 0.040 | 254.000 | 0.669 | 0.504 |
| **2014-2015:Time elapsed** | 0.098 | 0.030 | 254.000 | 3.218 | 0.002 |
| **2015-2016:Time elapsed** | -0.025 | 0.011 | 254.000 | -2.301 | 0.022 |
| **2016-2017:Time elapsed** | -0.011 | 0.117 | 254.000 | -0.090 | 0.928 |
| **2017-2018:Time elapsed** | -0.020 | 0.020 | 254.000 | -1.017 | 0.310 |

- **S3.3 Table : Maximal distance model .** Effect of the year in interaction with the timing in the season on the trip maximal distance (log-transformed).

***Model : log(Maximal distance) ~ Year * Time elapsed since the hatching peak date***

|  | **Value** | **Std.Error** | **DF** | **t-value** | **p-value** |
| --- | --- | --- | --- | --- | --- |
| **(Intercept)** | 3.197 | 0.161 | 254.000 | 19.892 | < 0.001 |
| **2011-2012** | 0.285 | 0.580 | 123.000 | 0.492 | 0.624 |
| **2014-2015** | -0.685 | 0.369 | 123.000 | -1.853 | 0.066 |
| **2015-2016** | -0.336 | 0.179 | 123.000 | -1.876 | 0.063 |
| **2016-2017** | -2.356 | 1.208 | 123.000 | -1.950 | 0.054 |
| **2017-2018** | 0.644 | 0.274 | 123.000 | 2.352 | 0.020 |
| **Time elapsed** | 0.029 | 0.010 | 254.000 | 2.993 | 0.003 |
| **2011-2012:Time elapsed** | 0.031 | 0.041 | 254.000 | 0.745 | 0.457 |
| **2014-2015:Time elapsed** | 0.092 | 0.031 | 254.000 | 2.985 | 0.003 |
| **2015-2016:Time elapsed** | -0.025 | 0.011 | 254.000 | -2.277 | 0.024 |
| **2016-2017:Time elapsed** | 0.064 | 0.119 | 254.000 | 0.540 | 0.590 |
| **2017-2018:Time elapsed** | -0.020 | 0.020 | 254.000 | -1.005 | 0.316 |

- **S3.4 Table : Bearing angle model .** Effect of the year and the year in interaction with the timing in the season on the heading between the most distal point and the colony.

***Model : Heading ~ Year + Year: Time elapsed since the hatching peak date***

|  | **Value** | **Std.Error** | **DF** | **t-value** | **p-value** |
| --- | --- | --- | --- | --- | --- |
| **(Intercept)** | 75.373 | 7.616 | 254.000 | 9.897 | < 0.001 |
| **2011-2012** | 24.914 | 24.673 | 123.000 | 1.010 | 0.315 |
| **2014-2015** | 14.870 | 16.774 | 123.000 | 0.881 | 0.377 |
| **2015-2016** | 0.926 | 8.527 | 123.000 | 0.109 | 0.914 |
| **2016-2017** | 127.262 | 53.911 | 123.000 | 2.361 | 0.020 |
| **2017-2018** | -5.858 | 12.872 | 123.000 | -0.455 | 0.650 |
| **2010-2011:Time elapsed** | -0.388 | 0.452 | 254.000 | -0.859 | 0.391 |
| **2011-2012:Time elapsed** | -1.122 | 1.663 | 254.000 | -0.675 | 0.500 |
| **2014-2015:Time elapsed** | 0.176 | 1.306 | 254.000 | 0.135 | 0.893 |
| **2015-2016:Time elapsed** | 0.666 | 0.262 | 254.000 | 2.541 | 0.012 |
| **2016-2017:Time elapsed** | -14.989 | 5.269 | 254.000 | -2.845 | 0.005 |
| **2017-2018:Time elapsed** | 0.363 | 0.804 | 254.000 | 0.451 | 0.652 |

- **S3.5 Table : Sea-ice concentration model .** Effect of the year in interaction with the timing in the season on the mean sea-ice concentration over the central phase of trip.

***Model : Sea-ice concentration (mean on the central phase of trip) ~ Year * Time elapsed since the hatching peak date***

|  | **Value** | **Std.Error** | **DF** | **t-value** | **p-value** |
| --- | --- | --- | --- | --- | --- |
| **(Intercept)** | 53.610 | 7.414 | 254.000 | 7.231 | < 0.001 |
| **2011-2012** | -28.080 | 28.963 | 123.000 | -0.970 | 0.339 |
| **2014-2015** | 58.685 | 17.616 | 123.000 | 3.331 | 0.001 |
| **2015-2016** | -17.850 | 8.239 | 123.000 | -2.167 | 0.032 |
| **2016-2017** | 46.390 | 58.264 | 123.000 | 0.796 | 0.427 |
| **2017-2018** | -67.535 | 12.754 | 123.000 | -5.295 | < 0.001 |
| **Time elapsed** | -1.827 | 0.441 | 254.000 | -4.144 | < 0.001 |
| **2011-2012:Time elapsed** | 3.658 | 2.061 | 254.000 | 1.774 | 0.077 |
| **2014-2015:Time elapsed** | 0.126 | 1.485 | 254.000 | 0.085 | 0.933 |
| **2015-2016:Time elapsed** | 0.492 | 0.509 | 254.000 | 0.966 | 0.335 |
| **2016-2017:Time elapsed** | 1.827 | 5.761 | 254.000 | 0.317 | 0.752 |
| **2017-2018:Time elapsed** | 5.002 | 0.921 | 254.000 | 5.430 | < 0.001 |

**S4 Table .** GLM results of the effect of the year in interaction with the timing in the season (“Time elapsed”, corresponding to the duration elapsed between each trip departure date and the peak hatching date) and the scale considered on the mean sea-ice concentration during each trip duration.

|  | **Estimate** | **Std. Error** | **t value** | **p-value** |
| --- | --- | --- | --- | --- |
| **(Intercept)** | 22.118 | 0.936 | 23.625 | < 0.001 |
| **2011-2012** | 71.911 | 8.114 | 8.866 | < 0.001 |
| **2014-2015** | 16.066 | 2.896 | 5.548 | < 0.001 |
| **2015-2016** | 1.380 | 1.006 | 1.372 | 0.171 |
| **2016-2017** | 23.560 | 10.092 | 2.335 | 0.020 |
| **2017-2018** | 4.915 | 1.925 | 2.553 | 0.011 |
| **Prospected_area** | 25.950 | 1.324 | 19.600 | < 0.001 |
| **Time elapsed** | -0.251 | 0.050 | -4.984 | < 0.001 |
| **2011-2012:Prospected_area** | -13.784 | 11.475 | -1.201 | 0.230 |
| **2014-2015:Prospected_area** | 13.440 | 4.095 | 3.282 | 0.001 |
| **2015-2016:Prospected_area** | -20.498 | 1.423 | -14.405 | < 0.001 |
| **2016-2017:Prospected_area** | 24.081 | 14.272 | 1.687 | 0.092 |
| **2017-2018:Prospected_area** | -12.357 | 2.722 | -4.539 | < 0.001 |
| **2011-2012:Time elapsed** | -2.236 | 0.515 | -4.341 | < 0.001 |
| **2014-2015:Time elapsed** | -0.062 | 0.215 | -0.290 | 0.772 |
| **2015-2016:Time elapsed** | -0.330 | 0.058 | -5.737 | < 0.001 |
| **2016-2017:Time elapsed** | -0.281 | 0.897 | 0.313 | 0.754 |
| **2017-2018:Time elapsed** | 0.135 | 0.130 | 1.0.39 | 0.299 |
| **Prospected_area :Time_elapsed** | -0.747 | 0.071 | -10.484 | < 0.001 |
| **2011-2012:Prospected_area:Time elapsed** | 0.202 | 0.729 | 0.278 | 0.782 |
| **2014-2015:Prospected_area:Time elapsed** | 0.906 | 0.304 | 2.980 | 0.003 |
| **2015-2016:Prospected_area:Time elapsed** | 0.825 | 0.081 | 10.141 | < 0.001 |
| **2016-2017:Prospected_area:Time elapsed** | 0.739 | 1.268 | 0.582 | 0.561 |
| **2017-2018:Prospected_area:Time elapsed** | 0.559 | 0.184 | 3.0.45 | 0.002 |

**S5 Tables .** GLMs results of the interaction between the year and the timing in the season on the NND (S5.1 Table), and GAMM results of the effect of the mean sea-ice concentrations and variations over each combination of successive trips (S5.2 Table).

- **S5.1 Table .** GLM results of the effect of the year in interaction with the timing in the season on the NND (log-transformed) during the central phase of birds’ trip.

| **Response variable** | **Predictor variables** | **Parametric coefficients** | | | **p-value** |
| --- | --- | --- | --- | --- | --- |
|  |  | **Estimate** | **SE** | **t** |  |
| **Log(Trip central phase NND)** | **(Intercept)** | 2.573 | 0.310 | 8.288 | < 0.001 |
|  | **2011-2012** | -1.379 | 2.690 | -0.513 | 0.609 |
|  | **2014-2015** | 0.357 | 0.960 | 0.372 | 0.710 |
|  | **2015-2016** | -0.958 | 0.334 | -2.870 | 0.004 |
|  | **2016-2017** | 2.589 | 3.346 | 0.773 | 0.440 |
|  | **2017-2018** | 1.458 | 0.638 | 2.284 | 0.023 |
|  | **Time elapsed** | 0.006 | 0.017 | 0.335 | 0.738 |
|  | **2011-2012:Time elapsed** | 0.147 | 0.171 | 0.861 | 0.390 |
|  | **2014-2015:Time elapsed** | -0.014 | 0.071 | -0.191 | 0.849 |
|  | **2015-2016:Time elapsed** | 0.004 | 0.019 | 0.195 | 0.846 |
|  | **2016-2017:Time elapsed** | -0.325 | 0.297 | -1.093 | 0.276 |
|  | **2017-2018:Time elapsed** | -0.138 | 0.043 | -3.214 | 0.001 |

- **S5.2 Table .** GAMM results of the effect of the mean and difference of the sea-ice concentrations over two trips, in the annually prospected area on the NND (log-transformed) during the central phase of birds’ trip.

| **Response variable** | **Predictor variables** | **Parametric coefficients** | | | **Approximate significance of smooth terms** | | | | **p-value** |  |
| --- | --- | --- | --- | --- | --- | --- | --- | --- | --- | --- |
|  |  | **Est.** | **SE** | **t** | | **Edf** | **F** |  | | |
| **Log(Trip central phase NND)** | **Intercept** | 2.053 | 0.065 | 31.64 | | - | - | < 0.001 | | |
|  | **Mean sea-ice concentration** | - | - | - | | 3.146 | 7.158 | < 0.001 | | |
|  | **Mean sea-ice concentration difference** | - | - | - | | 1.000 | 6.094 | 0.014 | | |

**S6 Table .** Repeatability in different species on several parameters (non-exhaustive review).

| **Authors** | **Year** | **Species** | **Parameters** | **Comparison groups** | **R** |
| --- | --- | --- | --- | --- | --- |
| **Janzen & Morjan** | 2001 | **Painted turtles (*Chrysemys picta*)** | Vegetation cover around nests at oviposition |  | **0.18** |
| **Dingemanse et al** | 2002 | **Great tits (*Parus major*)** | Exploratory behaviour | Female population Westerheide | **0.48** |
|  |  |  |  | Male population Westerheide | **0.27** |
|  |  |  |  | Female population Oosterhout | **0.66** |
|  |  |  |  | Male population Oosterhout | **0.46** |
| **Takahashi et al** | 2003 | **Adélie penguins (*Pygoscelis adeliae*)** | Time spent diving | Female (1995) | **0.27** |
|  |  |  |  | Female (1996) | **0.37** |
|  |  |  |  | Female (1997) | **0.12** |
|  |  |  |  | Female (1998) | **0.24** |
|  |  |  |  | Female (1999) | **0.23** |
|  |  |  |  | Male (1995) | **0.26** |
|  |  |  |  | Male (1996) | **0.12** |
|  |  |  |  | Male (1997) | **0.09** |
|  |  |  |  | Male (1998) | **0.12** |
|  |  |  |  | Male (1999) | **0.06** |
|  |  |  |  | Pair (1995) | **0.51** |
|  |  |  |  | Pair (1996) | **0.36** |
|  |  |  |  | Pair (1997) | **0.22** |
|  |  |  |  | Pair (1998) | **0.08** |
|  |  |  |  | Pair (1999) | **0.36** |
| **Nakagawa et al** | 2007 | **House sparrow (*Passer domesticus*)** | Incubation time | Male (2004) | **0.35** |
|  |  |  |  | Female (2004) | **0.11** |
|  |  |  |  | Male (2005) | **0.34** |
|  |  |  |  | Female (2005) | **0.34** |
|  |  |  | Feeding rate | Male (2004) | **0.46** |
|  |  |  |  | Female (2004) | **0.35** |
|  |  |  |  | Male (2005) | **0.44** |
|  |  |  |  | Female (2005) | **0.19** |
| **Biro & Ridgway** | 2008 | **Brook turtles (*Salvelinus fontinalis*)** | Proportion of time spent moving |  | **0.17** |
|  |  |  | Average search speed |  | **0.14** |
|  |  |  | Speed while moving during search |  | **0.16** |
|  |  |  | Pursuit distance |  | **0.31** |
| **Jenkins** | 2011 | **Merriam's kangaroo rats (*Dipodomys merriami*)** | Larder-hoarding propensity | Female | **0.03** |
|  |  |  |  | Male | **0.47** |
|  |  | **Ord's kangaroo rats (*Dipodomys ordii*)** |  | Female | **0.07** |
|  |  |  |  | Male | **0.86** |
| **Patrick et al** | 2014 | **Northern gannets (*Morus bassanus*)** | Longitude of distal point | Grassholm colony | **0.53** |
|  |  |  | Latitude of distal point |  | **0.54** |
|  |  |  | Departure angle |  | **0.71** |
|  |  |  | Trip duration |  | **0.00** |
|  |  |  | Total distance |  | **0.06** |
|  |  |  | Dive longitude |  | **0.84** |
|  |  |  | Dive latitude |  | **0.86** |
|  |  |  | Environmental conditions in dive sites (SST) |  | **0.46** |
|  |  |  | Environmental conditions in dive sites (chl a) |  | **0.77** |
|  |  |  | Environmental conditions in dive dites (Copepods) |  | **0.76** |
|  |  |  | Maximum dive depths |  | **0.18** |
|  |  |  | Dive shape |  | **0.18** |
|  |  |  | Longitude of distal point | Rouzic colony | **0.66** |
|  |  |  | Latitude of distal point |  | **0.57** |
|  |  |  | Departure angle |  | **0.55** |
|  |  |  | Trip duration |  | **0.00** |
|  |  |  | Total distance |  | **0.05** |
| **Lόpez-Lόpez et al** | 2014 | **Egyptian vulture (*Neophron percnopterus*)** | Departure date | Spring | **0.43** |
|  |  |  | Arrival date |  | **0.56** |
|  |  |  | Duration |  | **-0.18** |
|  |  |  | Linear distance |  | **0.87** |
|  |  |  | Cumulative distance |  | **0.33** |
|  |  |  | Speed |  | **-0.18** |
|  |  |  | Straightness |  | **0.43** |
|  |  |  | Latitude 30° |  | **0.30** |
|  |  |  | Latitude 24° |  | **0.16** |
|  |  |  | Departure date | Autumn | **0.71** |
|  |  |  | Arrival date |  | **0.70** |
|  |  |  | Duration |  | **0.12** |
|  |  |  | Linear distance |  | **0.71** |
|  |  |  | Cumulative distance |  | **0.13** |
|  |  |  | Speed |  | **0.35** |
|  |  |  | Straightness |  | **-0.12** |
|  |  |  | Latitude 30° |  | **0.00** |
|  |  |  | Latitude 24° |  | **0.03** |
| **Oppel et al** | 2015 | **Masked boobies (*Sula dactylatra*)** | Trip duration | St Helena island | **0.71** |
|  |  |  | Maximum distance |  | **0.48** |
|  |  |  | Total distance |  | **0.15** |
|  |  |  | Foraging direction |  | **0.08** |
|  |  |  | Trip duration | Ascension island | **0.63** |
|  |  |  | Maximum distance |  | **0.44** |
|  |  |  | Total distance |  | **0.52** |
|  |  |  | Foraging direction |  | **0.69** |
| **Patrick et al** | 2015 | **Northern gannets (Morus bassanus)** | Dive sites |  | **0.88** |
| **Potier et al** | 2015 | **Great cormorants (*Phalacrocorax carbo*)** | Departure angle |  | **0.33** |
|  |  |  | Latitude of distal point |  | **0.26** |
|  |  |  | Longitude of distal point |  | **0.08** |
|  |  |  | Trip duration |  | **0.33** |
|  |  |  | Maximal distance |  | **0.16** |
|  |  |  | Time spent underwater per trip |  | **0.08** |
|  |  |  | Time spent underwater per dive |  | **0.30** |
|  |  |  | Number of dive per trip |  | **0.14** |
| **Wakefield et al** | 2015 | **Northern gannets (*Morus bassanus*)** | δ15N | 2010 | **0.20** |
|  |  |  | δ13C |  | **0.37** |
|  |  |  | Trip duration |  | **0.04** |
|  |  |  | ARS scale |  | **0.01** |
|  |  |  | δ15N | 2011 | **0.35** |
|  |  |  | δ13C |  | **0.55** |
|  |  |  | Trip duration |  | **0.02** |
|  |  |  | ARS scale |  | **0.01** |
|  |  |  | Dive rate |  | **0.50** |
|  |  |  | Proportion of U-shaped dives |  | **0.07** |
|  |  |  | Mean dive depth |  | **0.38** |
|  |  |  | Mean max dive depth |  | **0.37** |
|  |  |  | δ15N | 2012 | **0.35** |
|  |  |  | δ13C |  | **0.18** |
|  |  |  | Trip duration |  | **0.01** |
|  |  |  | ARS scale |  | **0.02** |
|  |  |  | Dive rate |  | **0.04** |
|  |  |  | Proportion of U-shaped dives |  | **0.00** |
|  |  |  | Mean dive depth |  | **0.37** |
|  |  |  | Mean max dive depth |  | **0.29** |
|  |  |  | δ15N | Across years | **0.33** |
|  |  |  | δ13C |  | **0.03** |
|  |  |  | Trip duration |  | **0.01** |
|  |  |  | ARS scale |  | **0.02** |
|  |  |  | Dive rate |  | **0.43** |
|  |  |  | Proportion of U-shaped dives |  | **0.00** |
|  |  |  | Mean dive depth |  | **0.36** |
|  |  |  | Mean max dive depth |  | **0.21** |
| **Vardanis et al** | 2016 | **Osprey (*Pandion haliaetus*)** | Longitude of route (Europe) | Spring | **0.88** |
|  |  |  | Longitude of route (Mediterranean) |  | **0.87** |
|  |  |  | Longitude of route (Sahara) |  | **0.54** |
|  |  |  | Timing of migration (Europe) |  | **0.17** |
|  |  |  | Timing of migration (Mediterranean) |  | **0.19** |
|  |  |  | Timing of migration (Sahara) |  | **0.04** |
|  |  |  | Duration |  | **0.35** |
|  |  |  | Longitude of route (Europe) | Autumn | **0.99** |
|  |  |  | Longitude of route (Mediterranean) |  | **0.81** |
|  |  |  | Longitude of route (Sahara) |  | **0.04** |
|  |  |  | Timing of migration (Europe) |  | **0.07** |
|  |  |  | Timing of migration (Mediterranean) |  | **0.37** |
|  |  |  | Timing of migration (Sahara) |  | **0.38** |
|  |  |  | Duration |  | **0.26** |
|  |  | **Marsh harrier (*Circus aeruginosus*)** | Longitude of route (Europe) | Spring | **0.06** |
|  |  |  | Longitude of route (Mediterranean) |  | **0.41** |
|  |  |  | Longitude of route (Sahara) |  | **0.09** |
|  |  |  | Timing of migration (Europe) |  | **0.35** |
|  |  |  | Timing of migration (Mediterranean) |  | **0.55** |
|  |  |  | Timing of migration (Sahara |  | **0.60** |
|  |  |  | Duration |  | **0.92** |
|  |  |  | Longitude of route (Europe) | Autumn | **-0.13** |
|  |  |  | Longitude of route (Mediterranean) |  | **-0.11** |
|  |  |  | Longitude of route (Sahara) |  | **-0.15** |
|  |  |  | Timing of migration (Europe) |  | **0.63** |
|  |  |  | Timing of migration (Mediterranean) |  | **0.32** |
|  |  |  | Timing of migration (Sahara) |  | **0.81** |
|  |  |  | Duration |  | **0.74** |
| **Patrick et al** | 2017 | **Northern gannets (*Morus bassanus*)** | Exploration-exploitation strategy |  | **0.40** |
|  |  |  | Time in patch |  | **0.25** |
|  |  |  | Foraging effort |  | **0.21** |
|  |  |  | Size of patch |  | **0.71** |
|  |  |  | Number of patches |  | **0.48** |
| **Votier et al** | 2017 | **Northern gannets (*Morus bassanus*)** | Longitude of distal point | Breeders | **0.51** |
|  |  |  | Latitude of distal point |  | **0.34** |
|  |  |  | Total distance |  | **0.11** |
|  |  |  | Maximal distance |  | **0.15** |
|  |  |  | Longitude of distal point | Failed breeders | **0.42** |
|  |  |  | Latitude of distal point |  | **0.00** |
|  |  |  | Total distance |  | **0.01** |
|  |  |  | Maximal distance |  | **0.00** |
|  |  |  | Longitude of distal point | Immatures | **0.00** |
|  |  |  | Latitude of distal point |  | **0.00** |
|  |  |  | Total distance |  | **0.00** |
|  |  |  | Maximal distance |  | **0.00** |
| **Delord et al** | 2019 | **Grey Petrels (*Procellaria cinerea*)** | Date of departure from breding colony (spring) |  | **0.44** |
|  |  |  | Date of arrival at non-breeding area |  | **0.55** |
|  |  |  | Date of departure from non-breeding area |  | **0.70** |
|  |  |  | Date of arrival at breeding colony (autumn) |  | **0.41** |
|  |  |  | Time spent in non-breeding areas |  | **0.63** |
|  |  |  | Duration of outward migration |  | **0.87** |
|  |  |  | Duration of inward migration |  | **0.96** |
|  |  |  | Anomaly in longitude of centroid 50% kernel UD |  | **0.00** |
|  |  |  | Anomaly in latitude of centroid 50% kernel UD |  | **0.00** |
|  |  |  | 50% kernel UD overlap for non-breeding period |  | **0.41** |
|  |  |  | 95% kernel UD overlap for non-breeding period |  | **0.13** |
|  |  |  | Maximum range distance during non-breeding period |  | **0.20** |
|  |  |  | Ratio 50% kernel UD individual/population |  | **0.46** |
|  |  |  | % of time on water during daylight during the non-breeding period |  | **0.77** |
|  |  |  | % of time on water during darkness during the non-breeding period |  | **0.75** |
|  |  |  | % of time on water during 24h during the non-breeding period |  | **0.84** |
| **Traisnel & Pichegru** | 2019 | **African penguins (*Spheniscus demersus*)** | Maximum distance |  | **0.33** |
|  |  |  | Trip duration |  | **0.33** |
|  |  |  | Path length |  | **0.31** |
|  |  |  | Straightness |  | **0.14** |
|  |  |  | Bearing |  | **0.55** |
| **Michelot et al** |  | **Adélie penguins (*Pygoscelis adeliae*)** | **Trip duration** |  | **0.40** |
|  |  |  | **Total distance** |  | **0.28** |
|  |  |  | **Maximal distance** |  | **0.26** |
|  |  |  | **Bearing angle** |  | **0.38** |
|  |  |  | **Environmental conditions in foraging areas** |  | **0.17** |

**References (that are not already cited in the article)**

Biro PA, Ridgway MS (2008) Repeatability of foraging tactics in young brook trout, *Salvelinus fontinalis*. The Canadian field-naturalist 122(1):40-44

Delord K, Barbraud C, Pinaud D, Ruault S, Patrick SC, Weimerskirch H (2019) Individual consistency in the non-breeding behavior of a long-distance migrant seabird, the Grey Petrel Procellaria cinerea. Marine Ornithology 47:93-103

Janzen FJ, Morjan CL (2001) Repeatability of microenvironment-specific nesting behaviour in a turtle with environmental sex determination. Animal Behaviour 62(1):73-82

Jenkins SH (2011) Sex differences in repeatability of food-hoarding behaviour of kangaroo rats. Animal Behaviour 81(6):1155-1162

López-López P, García-Ripollés C, Urios V (2014) Individual repeatability in timing and spatial flexibility of migration routes of trans-Saharan migratory raptors. Current Zoology 60(5):642-652

Nakagawa S, Gillespie DOS, Hatchwell BJ, Burke T (2007) Predictable males and unpredictable females: sex difference in repeatability of parental care in a wild bird population. Journal of evolutionary biology 20(5):1674-1681

Oppel S, Beard A, Fox D, Mackley E, Leat E, Henry L, ... Weber N (2015) Foraging distribution of a tropical seabird supports Ashmole’s hypothesis of population regulation. Behavioral Ecology and Sociobiology 69(6):915-926

Patrick SC, Bearhop S, Bodey TW, Grecian WJ, Hamer KC, Lee J, Votier SC (2015) Individual seabirds show consistent foraging strategies in response to predictable fisheries discards. Journal of Avian Biology 46(5):431-440

Patrick SC, Pinaud D, Weimerskirch H (2017) Boldness predicts an individual's position along an exploration–exploitation foraging trade‐off. Journal of Animal Ecology 86(5):1257-1268

Vardanis Y, Nilsson JÅ, Klaassen RH, Strandberg R, Alerstam T (2016) Consistency in long-distance bird migration: contrasting patterns in time and space for two raptors. Animal Behaviour 113:177-187
